# Spectroscopic, Electrochemical and DFT Studies of Phosphorescent Homoleptic Cyclometalated Iridium(III) Complexes Based on Substituted 4-Fluorophenylvinyl- and 4-Methoxyphenylvinylquinolines

**DOI:** 10.3390/ma10101061

**Published:** 2017-09-21

**Authors:** Adewale O. Adeloye, Malose J. Mphahlele, Abolanle S. Adekunle, Lydia Rhyman, Ponnadurai Ramasami

**Affiliations:** 1 Department of Chemistry, College of Science, Engineering and Technology, University of South Africa, Private Bag X6, Florida 1710, South Africa; mphahmj@unisa.ac.za; 2 Department of Chemistry, Faculty of Science, Obafemi Awolowo University, Ile-Ife, Nigeria; aadekunle@oauife.edu.ng; 3 Computational Chemistry Group, Department of Chemistry, Faculty of Science, University of Mauritius, Réduit 80837, Mauritius; lyd.rhyman@gmail.com (L.R.); ramasami@uom.ac.mu (P.R.)

**Keywords:** cyclometalated iridium(III) complexes, 4-fluorophenylvinylquinoline, 4-methoxyphenylvinylquinoline, photophysical properties, phosphorescence, electrochemistry, DFT

## Abstract

This study reports the synthesis and comparative investigation of the substituent effects of a new series of highly luminescent homoleptic tris-cyclometalated iridium(III) complexes of the type [Ir(N^˄^C)_3_]. These are based on two ligand type derivatives comprising of 4-fluorophenylvinylquinolines and 4-methoxyphenylvinylquinolines with electron-donating and/or electron-withdrawing groups as aryl substituents at 2-position. The structures of the ligands and their complexes were characterized by means of FT-IR, UV-Vis and NMR spectrometry complemented with photoluminescence and cyclic voltammetry. The photophysical properties of 2-aryl-4-(4-fluorophenylvinyl)quinoline and its corresponding complex were also studied using the density functional theory method. The photoluminescent properties of the ligands and the corresponding complexes showed high fluorescent intensities and quantum yields in solvents of different polarities. The photoluminescence spectra of the complexes in solid film, showed common transmission curves at longer wavelengths maximum (λ_em_ = 697 nm) possibly originating from the interference of scattered light of higher-order transmission of monochromators.

## 1. Introduction

In the past few years, luminescent cyclometalated iridium(III) complexes have continued to attract significant attention. The studies involving the photophysical and electrochemical properties of iridium coordination and organometallic complexes have gathered much interest. The reasons for such an interest are due to their strong metal-ligand bonding interactions as well as their ability to emit light from both singlet and triplet excitons leading to high chemical photostability, which allows continuous exposure of the complexes to irradiation, long emission lifetimes, and large Stokes’ shifts [1,2,3,4,5,6,7,8,9,10,11,12,13]. The tuning of the second-order nonlinear optical (NLO) response of the unusual family of 3D chromophores of certain cyclometallated Ir(III) complexes with appropriate ligand substitution has allowed contribution to their interesting luminescent properties [14]. A correlated investigation of the photoluminescent and electroluminescent properties at different bias voltages of iridium(III) ionic transition metals complexes (Ir-iTMC), for example, has been shown to be dependent on the applied electric field. Increased bias voltage leads to Electroluminescent (EL) spectrum dominated by red-shifted components that are related to the morphological rearrangement of the materials due to enhanced local electric field. The overall effect is the contribution to degradation and changes in the spectral behavior of the electroluminescent and photoluminescent decay times [14,15,16,17,18]. The development of pure-blue-to-deep-blue-emitting ionic phosphors is an ultimate challenge for full-colour displays and white-light sources in the field of optoelectronics. However, it has been reported that the control of the frontier orbital energy level (HOMO-LUMO, Highest Occupied Molecular Orbital-Lowest Unoccupied Molecular Orbital) is the sole method to achieve better blue phosphorescent iridium complexes by appropriate ligand selection and the introduction of adequate substituents [19]. 

Based on the synthesis of cyclometalated Ir(ppy)_3_ (where ppy represents 2-phenylpyridine), which has been extensively investigated as a green material for Organic Light Emitting Diodes (OLEDs), a strong emission originates from the triplet excited states having both the π→π* and metal-to-ligand-charge-transfer (MLCT) characteristics. Several methods have been employed in order to tune the emission to the red region of the spectrum. The elongation of conjugative bonds in molecules, replacement of one CH– group at the pyridyl ring of phenylpyridine ligand by nitrogen, doping of cyclometalated iridium complexes into a host thus given control of the intermolecular interaction, introduction of dendrimers due to their three-dimensional hyper branched structural types into the molecular structures and the addition of either electron-donating or electron-withdrawing functional groups to the pyridyl ring and the phenyl ring, respectively, were among the methods used for wavelength enhancement [20,21,22,23,24,25,26,27,28]. 

In this paper, we report the synthesis, characterization and comparative studies of eight novel highly luminescent 4-fluorophenylvinylquinolines (**2a**–**d**) and 4-methoxyphenylvinylquinolines (**3a**–**d**) ligands containing both electron donating and withdrawing groups particularly as aryl-substituents at 2-position of the quinoline scaffold and their corresponding homoleptic cyclometalated iridium(III) complexes of the type [Ir(N^∧^C)_3_]. The molecular rearrangement of the different substituents on quinoline ligands reflects the general changes in the photophysical and electrochemical properties of the compounds. We complemented this study using the density functional theory (DFT) method on one of the ligands, namely 2-aryl-4-(4-fluorophenylvinyl)quinoline and its corresponding complex.

## 2. Results

### 2.1. Synthesis

**Scheme 1:**
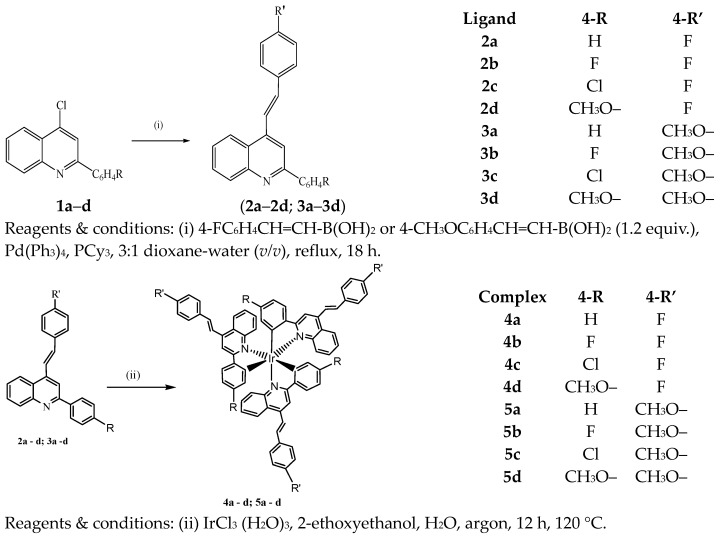
Suzuki cross-coupling of the 2-aryl-4-chloroquinolines **1a**–**d** with arylvinylboronic acids.

The synthesis of the reported compounds first involves the use of the 2-aryl-4-chloroquinolines (**1a**–**d**) as substrates for palladium-catalyzed Suzuki cross-coupling with 4-(fluorophenylvinyl)boronic acid and 4-(methoxyphenylvinyl)boronic acid (1.2 equiv.) as coupling partners in the presence of Pd(PPh_3_)_4_–PCy_3_ catalyst complex as Pd(0) source and potassium carbonate as a base in 3:1 dioxane-water mixture (*v/v*) under reflux (Scheme 1) [29]. The prepared 2-aryl-4-(4-fluorophenylvinyl)quinolines (**2a**–**d**) and 2-aryl-4-(4-methoxyphenylvinyl)quinolines (**3a**–**d**) were used in this investigation as models for comparative studies in the electronic absorption and emission properties, and a further electrochemical studies for the corresponding homoleptic Ir(III) complexes of the type [Ir(N^˄^C)_3_], coded as **4a**–**d** and **5a**–**d**, respectively. The structure of the prepared complexes was characterized by means of ^1^H NMR spectroscopy and elemental (C/H/N) analysis. The coordinates for the optimised structures of the ligand **2a** and the corresponding complex **4a** in the gas phase are provided as Supplementary Materials (Tables S1 and S2, respectively). Table 1 summarizes the selected bond lengths (Ir–N and Ir–C) of the complex (**4a**). The literature values of these bond lengths of compounds with quinoline moiety are also included in the table, and it is observed that there is a good comparison.

### 2.2. Electronic Absorption and Luminescence Spectra of Ligands

#### 2.2.1. Electronic Absorption of Ligands

**Figure 1:**
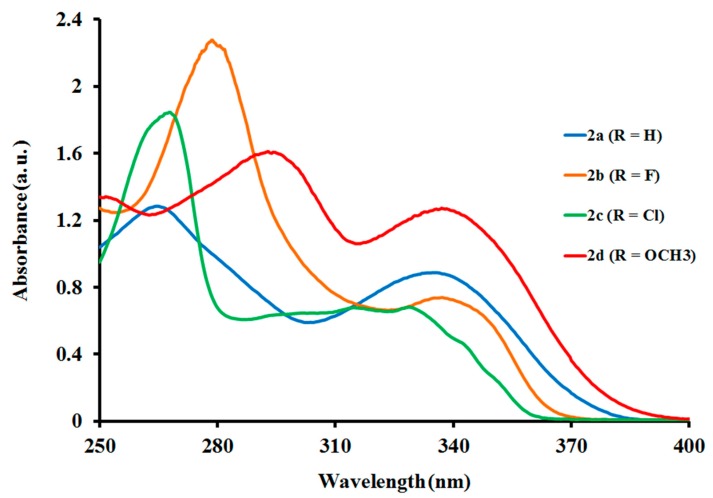
UV-Vis Absorption spectra of (**2a**–**d**) in chloroform (concentration 1.0 × 10^−3^ mol/L).

**Figure 2:**
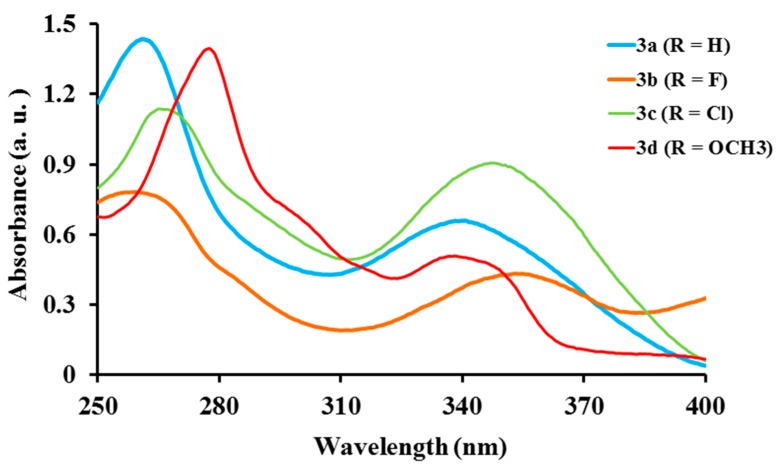
UV-Vis absorption spectra of **3a**–**d** in chloroform (concentration 1.0 × 10^−3^ mol/L).

As a prelude to compounds with potential photoelectronic properties, the absorption spectra of the ligands were investigated in solution at room temperature (RT). The electronic absorption of the compounds **2a**–**d** and **3a**–**d** measured in chloroform are shown in Figure 1 and Figure 2 and summarized in Table S3 (see Supplementary Materials). The strong absorption maxima wavelength bands in the region λ_abs_ = 261–297 nm in the ultraviolet region areas were assigned to the spin-allowed ligand-centered (^1^LC) π→π* transitions and the bands in the region λ_abs_ = 320–354 nm also assigned to the π→π* intramolecular charge transitions (ICT), which possibly originate from further conjugation made by the electron donor and electron acceptor species present in the cyclometalated ligands. The types and positions of the substituents were found to have strong effects on the molar extinction coefficients (ε) and wavelengths (λ_abs_) of the compounds [31,32,33]. By comparison, ligands **2a** and **3a** with a phenyl substituent at position 2 of the quinoline framework showed no apparent change in the molar extinction coefficient, as the wavelengths almost close to each other. Clear distinctions of the molar extinction coefficient were, however, observed with the 2-aryl-4-(4-fluorostyryl)quinolines **2b**–**d** and 2-aryl-4-(4-methoxystyryl)quinolines **3b**–**d** substituted at the *para*-positon of the 2-aryl ring with a strong electron withdrawing halogen atom (F, Cl) and strong electron donating methoxy group. A red-shift in wavelength maxima was observed **2b** (λ_abs_ = 278 nm) and **2d** (λ_abs_ = 293 nm) substituted with 2-(4-fluorophenyl)- and 2-(4-methoxyphenyl)- group, respectively. On the other hand, a blue-shift in wavelength to λ_abs_ = 267 nm was observed for **2c** substituted with a 2-(4-chlorophenyl) group. By comparison, in the red region of absorption, compounds **2a**–**c** and **3a**–**c** showed a corresponding steady increase in wavelength maxima at (λ_abs_ = 335, 337 and 328 nm) and (λ_abs_ = 340, 354 and 348 nm), respectively. No apparent change was observed in the wavelength maxima for **2d** and **3d** at (λ_abs_ = 338 nm). DFT computation in chloroform indicates that the most intense peak appears at λ_abs_ = 362 nm (experiment, λ_abs_ = 335 nm) with an oscillator strength of 0.595, and this is assigned to HOMO to LUMO transition. The HOMO-LUMO gap of ligand (**2a**) is 3.82 eV in chloroform. Similar patterns in the absorption wavelengths were observed in the analogues’ derivatives **3b**, **3c** and **3d** at λ_max_ = 354, 348 and 338 nm, respectively. However, significant changes in spectral pattern and reduced molar extinction coefficient generally characterized the ligands **3b**, **3c** and **3d** than those of **2b**, **2c**, **2d** with exception in **3c**, which showed significant hyperchromic effect. It has been reported that an increase in the wavelength as observed for the 4-methoxyphenyl substituents plays a useful role in the energy gap determination between the highest occupied molecular orbital (HOMO) and the lowest unoccupied molecular orbital (LUMO) [28]. It is well known that consideration of the electronic effects has been shown to have a significant influence on orbital energies and the relative ease with which electron-withdrawing (e.g., –F, –CF_3_) and electron-donating (e.g., –(CH_3_)_3_, –OCH_3_) groups can be incorporated into the ligand structure. Accordingly, electron-withdrawing substituents tend to stabilize the HOMO by removing electron density from the metal, whereas donating groups have an inverse effect [34,35,36,37,38]. This relationship is complicated by the fact that electron withdrawing groups may also lower the energy of the LUMO (i.e., increasing the electron affinity of the parent ligand).

#### 2.2.2. Photoluminescence Properties of Ligands

**Figure 3:**
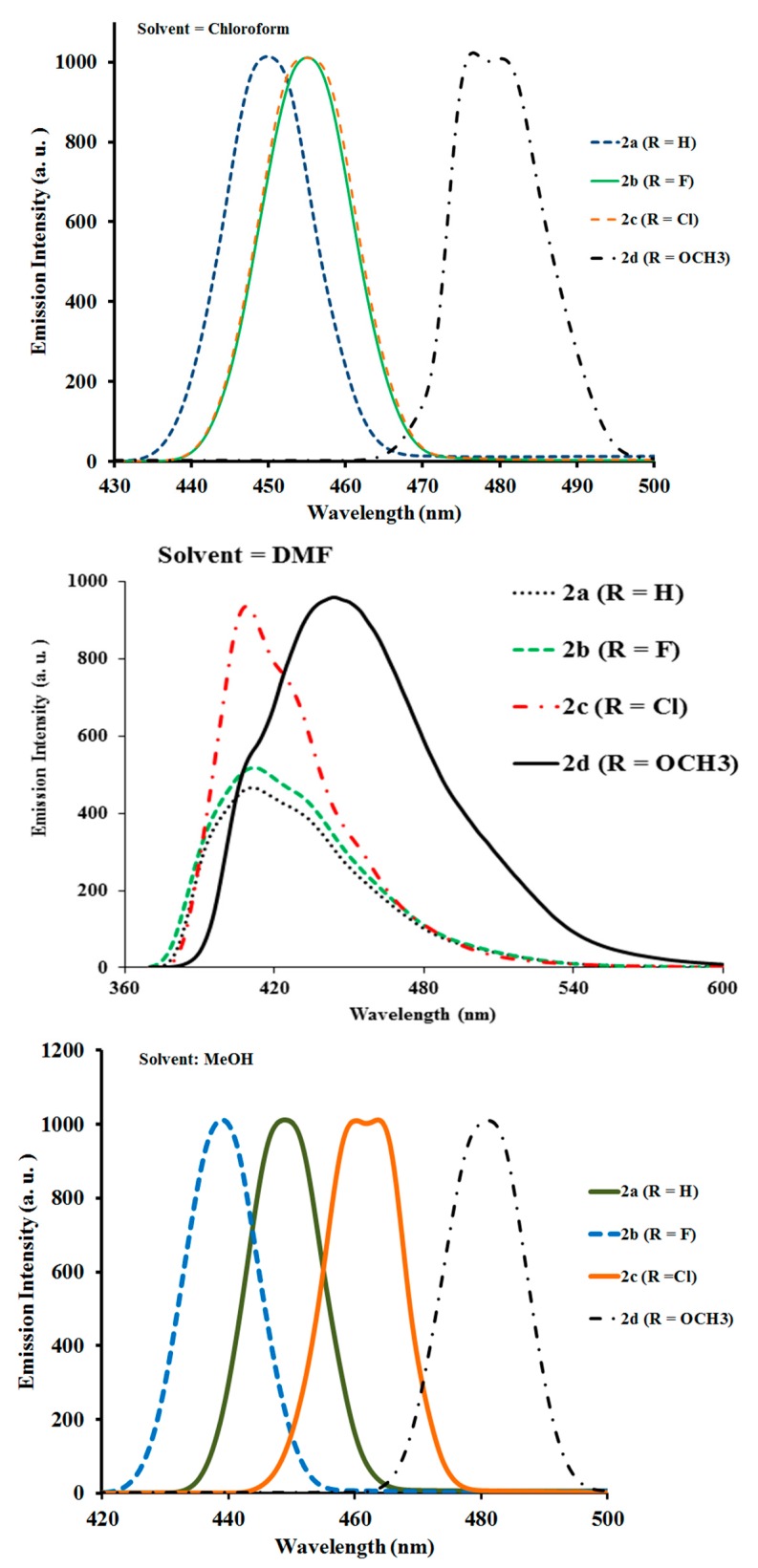
Fluorescence emission spectra of **2a**–**d** (concentration 2.0 × 10^−7^ mol/L) at RT (room temperature).

**Figure 4:**
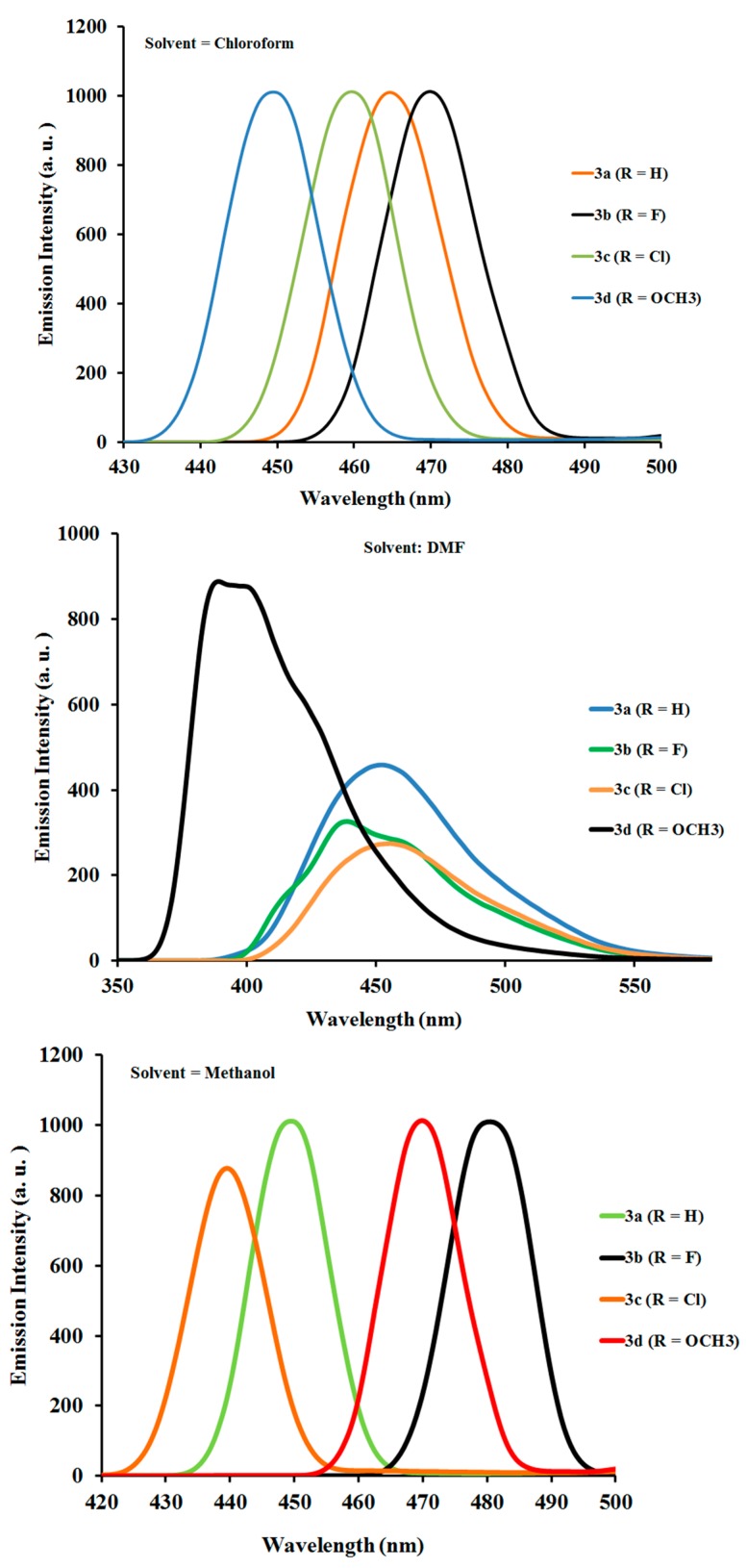
Fluorescence emission spectra of **3a**–**d** (concentration 2.0 × 10^−7^ mol/L) at RT.

The emission properties of the ligands were investigated in three different solvents of varying polarities at excitation wavelengths in the region λ_ex_ = 350–385 nm. Highly intense photoluminescence (PL) with a similar pattern characterized by single emission bands were observed for the ligands **2a**–**d** and **3a**–**d** in chloroform and methanol with λ_em_ values located in the regions 449–478 nm and 455–470 nm, respectively (Figure 3 and Figure 4). These values in dimethyl formamide solution are located in the region λ_em_ at 406–442 nm (see Figure 3b) for **2a**–**d** and λ_em_ 389–454 nm for **3a**–**d** (refer to Figure 4b). The effects of the position or type of substituents on quinoline framework were particularly observed in the 2-(4-halogenophenyl)-4-(4-fluorostyryl)quinolines **2b** and **2d**, and the analogous 2-(4-halogenophenyl)-4-(4-methoxystyryl)quinolines **3b** and **3d**. In chloroform or methanol solutions, **2b** with a combination of 2-(4-fluorophenyl)- and 4-(4-fluorostyryl) moieties was found to exhibit lower emission value at λ_em_ = 454 and 439 nm, respectively. Compound **3d** with a combination of the 2-(4-methoxyphenyl)- and 4-(4-methoxystyryl) groups was found to emit at λ_em_ = 455 and 470 nm. Likewise, a combination of 2-(4-methoxyphenyl)- and 4-(4-fluorostyryl) groups in **2b** or 2-(4-fluorophenyl)- and 4-(4-methoxystyryl) groups in **3b** resulted in the highest emission wavelength at λ_em_ = 478 and 482 nm or λ_em_ = 470 and 481 nm, respectively [26]. Interestingly, the nature and position of the substituents on the aromatic rings did not show any significant effect on the wavelength shifts between **2d** and **3b** in the polar protic methanol. Accordingly, the quinoline framework is expected to be made more electron-deficient as a result of the formation of strong solute–solvent interaction through intermolecular hydrogen bond with N-1 as expected from the strong protic methanol [28]. On the other hand, in the DMF solution, the effect of the electron-withdrawing groups increases the emission intensity steadily between the 4–FC_6_H_4_- and the 4–ClC_6_H_4_- group and the trend is as follows: **2c** > **2b** > **2a**. However, this effect is of a lesser extent when compared to the electron-donating methoxy group in **2d** with a bathochromic shift in emission wavelength (Figure 3b). On the other hand, the emission intensities of compounds **3a**–**d** were significantly reduced and followed the trend **3a** > **3b** > **3c** with a blue-shift in emission wavelength of about 53 nm in **3d** compared to **2d** (Figure 4b). 

### 2.3. Electronic Absorption and Luminescence Spectra of Complexes

#### 2.3.1. Electronic Absorption of Complexes

**Figure 5:**
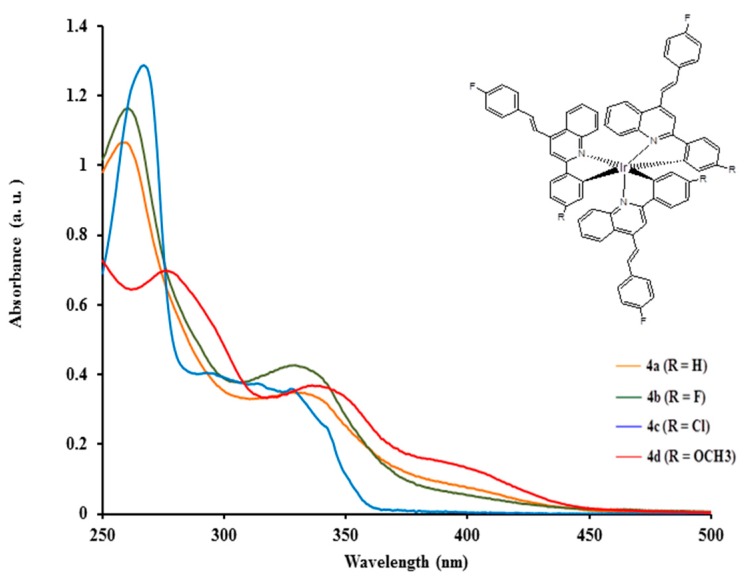
UV-Vis Absorption spectral of Ir(III) complexes **4a**–**d** in methanol (concentration 1.0 × 10^−3^ mol/L).

**Figure 6:**
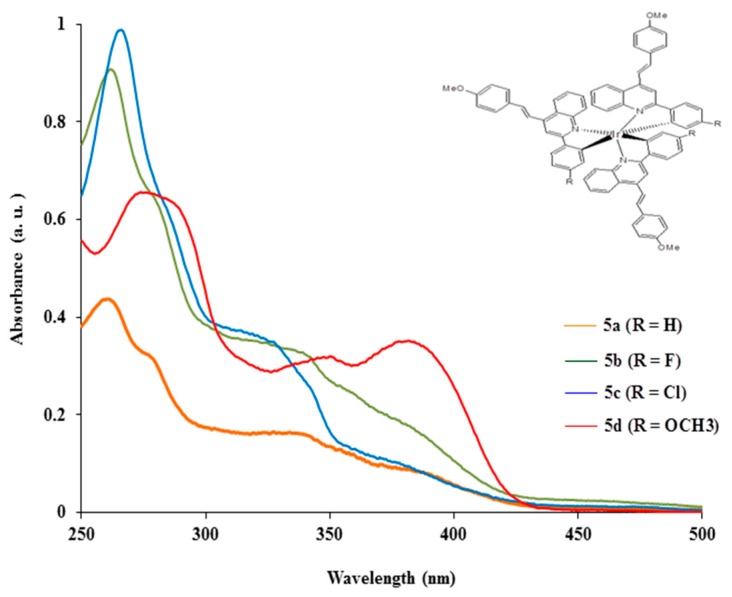
UV-Vis Absorption spectral of Ir(III) complexes of **5a**–**d** in methanol (concentration 1.0 × 10^−3^ mol/L).

The electronic absorption spectra of the homoleptic Ir(III) cyclometalated complexes **4a**–**d** and **5a**–**d** obtained from ligands **2a**–**d** and **3a**–**d** and recorded at room temperature in methanolic solution are shown in Figure 5 and Figure 6, respectively, and the corresponding data is summarized in Table S4 in the Supplementary Materials. All of the complexes showed intense spin-allowed intraligand (^1^IL) absorption bands at the higher energy region λ_max_ = 261–300 nm (ε on the order of 10^4^ dm^3^ mol^−1^ cm^−1^), which are attributed to the π→π* transitions and the intramolecular charge transfer transitions due to derivatized quinoline framework. The less intense low-lying absorption bands were in the range λ_max_ 335–397 nm for Ir(III)-(**4a**–**d**) and λ_max_ 348–384 nm for Ir(III)-(**5a**–**d**) complexes. These bands are attributed to the spin-allowed metal-to-ligand charge transfer (^1^MLCT) (dπ(Ir)→π*(N^˄^C) transitions, although assumed to be possibly mixed to a certain extent, with ligand-centered π→π* transitions to form metal-to-ligand-ligand charge transfer (MLLCT) [39]. On the other hand, the spin-forbidden metal-to-ligand charge transfer band (^3^MLCT) transitions appear as weak shoulder bands at lower energy absorption in the λ_max_ region 450–500 nm, which may be attributed to (dπ(Ir)→π*(N^˄^C) transitions [40]. The most intense peak of complex (**4a**) in methanol is observed at λ_max_ = 378 nm or 3.28 eV with an oscillator strength of 0.7374, and this is assigned mostly to HOMO-4 to LUMO transition. The HOMO-LUMO gap is 2.99 eV, and this gap is in agreement with analogous iridium complexes reported in the literature [41]. 

As observed in Figure 5, the introduction of 4-(4-fluorophenylvinyl) group on the quinoline framework and the presence of a 2-(4-halogenophenyl) ring lead to increased molar extinction coefficients at the higher energy region of the spectra as compared to those for 2-phenyl- and 2-(4-methoxyphenyl)substituted derivatives in the trend **4c** > **4b** > **4a** > **4d**. The absorption wavelength maxima for these complexes tend towards the lower energy region and the trend is reversed as follows: **4d** > **4a** > **4b** > **4c**. Complex **4c** with a 2-(4-chlorophenyl) substituted 4-(4-fluorostyryl)quinoline framework does not exhibit appreciable MLCT absorption band in the wavelength region longer than λ_max_ = 360 nm. Complexes **4a**, **4b** and **4d**, on the other hand, have absorption extended to about λ_max_ = 445 nm, which may be due to the forbidden ^3^MLCT transition of the complexes. It is well known in the literature that molecules with intramolecular donor–acceptor (DA) systems exhibit bathochromic shifts of both absorption and emission spectra [21]. A low ^1^MLCT absorption band was recorded for complex **4c**, and the reason for this anomalous behavior is not clear to us at this stage. The characteristics of the absorption wavelengths were strongly influenced when the substituent on the 4-position of the quinoline scaffold is the 4-methoxyphenylvinyl functionality with variations in the *para*-position of the 2-aryl group incorporating the electron-donating or electron-withdrawing substituents as shown in Figure 6. The absorption spectra of complexes **5a**–**d** when compared to those of **4a**–**d** counterparts (see Figure 5) showed absorption wavelengths for **5b** and **5c** containing the 4-(halogenophenyl) rings at the 2-position. These two complexes maintained their higher molar extinction coefficient values at the higher energy levels as compared to other complexes containing the 2-phenyl or 2-(4-methoxyphenyl) substituted quinoline scaffolds with the following trend **5c** > **5b** > **5d** > **5a**. However, in a similar fashion, as the absorption bands tend towards the lower energy region of spectra, the molar extinction coefficients of the halogenophenyl group decrease steadily in the trend **5d** > **5b** > **5c** > **5a**. Clear and significant reduction in molar extinction coefficient was observed for **5a** (ε = 0.27 × 10^4^ M^−1^ cm^−1^) having only phenyl ring at the 2-position of the quinoline framework as compared to **5d** with 4–OCH_3_ (ε = 0.61 × 10^4^ M^−1^ cm^−1^). It has previously been established that an electron-donating group can influence the highest occupied molecular orbital (HOMO) and/or the lowest unoccupied molecular orbital (LUMO) level as well as ^1^MLCT state of the iridium(III) complex [32].

#### 2.3.2. Photoluminescent Properties of Complexes

**Figure 7:**
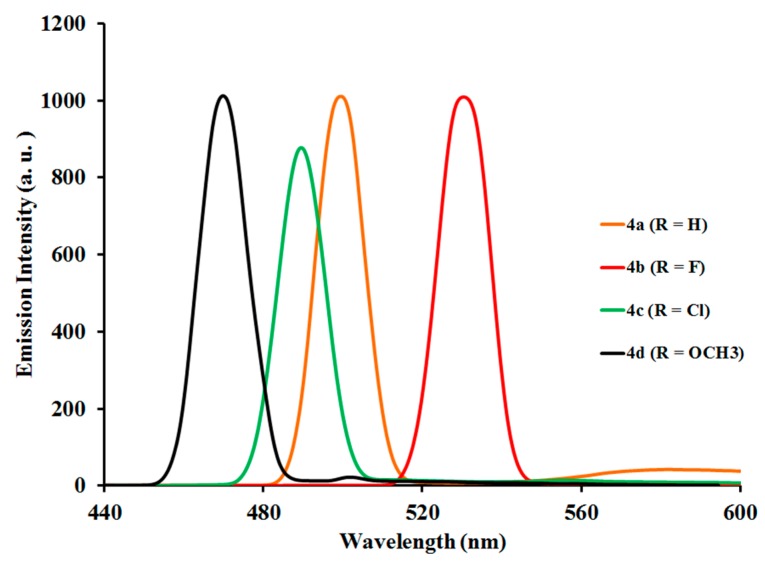
Phosphorescence emission spectra of (Ir(**4a**–**d**)_3_) at λ_ex_ = 350 nm in methanol (concentration 2.0 × 10^−7^ mol/L) at RT.

**Figure 8:**
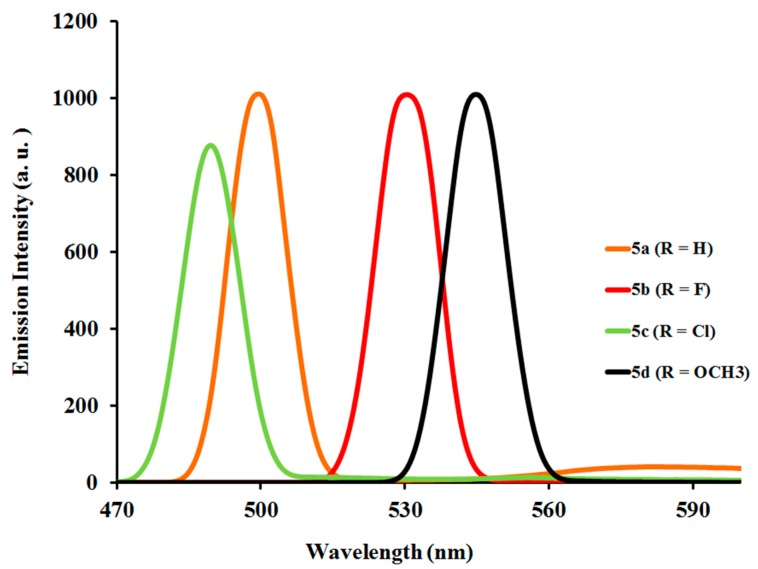
Phosphorescence emission spectra of (Ir(**5a**–**d**)_3_) at λ_ex_ = 350 nm in methanol (concentration 2.0 × 10^−7^ mol/L) at RT.

**Figure 9:**
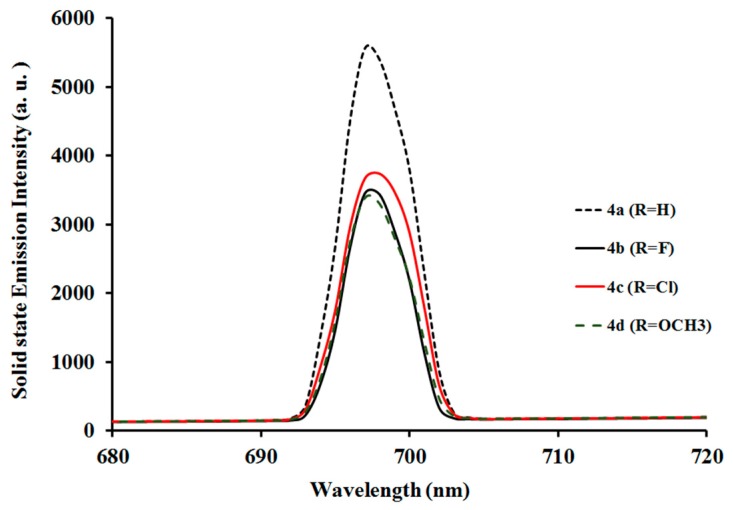
Solid state phosphorescence emission spectra of (Ir(**4a**–**d**)_3_) at λ_ex_ = 350 nm at RT.

**Figure 10:**
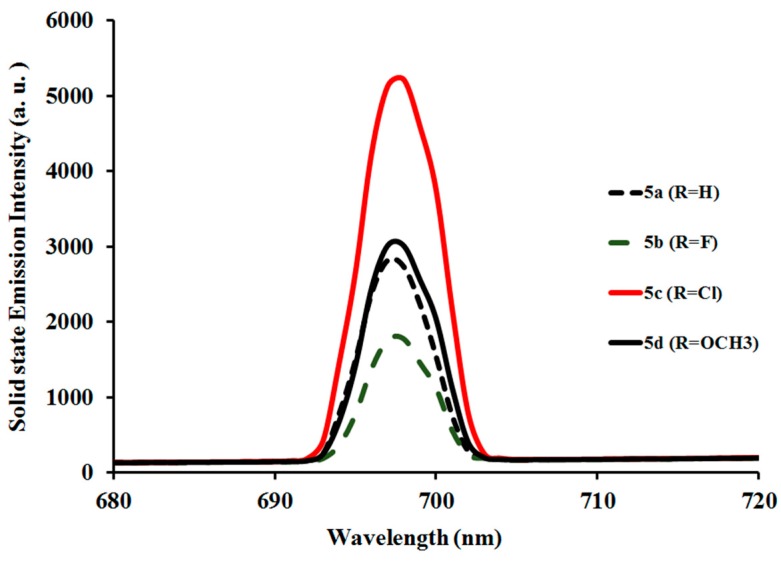
Solid state phosphorescence emission spectra of (Ir(**5a**–**d**)_3_) at λ_ex_ = 350 nm at RT.

Photoluminescent properties of the complexes **4a**–**d** and **5a**–**d** were investigated both in methanolic solution as well as in the solid state at room temperature. Figure 7, Figure 8, Figure 9 and Figure 10 show the luminescence spectra of the Ir complexes and summary of data reported in Table S4 in the Supplementary Materials. All of these complexes emit intense phosphorescence bands with λ_em_ values ranging from 470 nm to 545 nm in solution, which corresponds to blue-green light [39,40,41]. In comparison to complexes containing 4-(4-fluorophenylvinyl)quinoline as the common substituent group as shown in Figure 7, complex **4b** having 2-(4-fluorophenyl) group exhibits the highest emission wavelength at λ_em_ = 530 nm, followed by the 2-phenyl substituted complex **4a** at λ_em_ = 499 nm. A further blue-shift in wavelengths was observed at λ_em_ = 489 nm or at λ_em_ = 470 nm for **4c** or **4d** bearing the 2-(4-chlorophenyl) or 2-(4-methoxyphenyl) group, respectively. As shown in Figure 8, no appreciable changes in wavelengths were observed for the 2-phenyl- **5a**, 2-(4-flurophenyl) **5b** and 2-(4-chlorophenyl) substituted derivative **5c**. However, moderate red-shift in wavelength at λ_em_ = 545 nm was observed for **5d** having strongly electron-donating 2-(4-methoxyphenyl) and 4-(4-methoxystyryl) groups on the quinolone framework. As previously accounted for in the ligands, it may be assumed possibly that strong solute–solvent interaction through intermolecular hydrogen bond with N-1 as expected from the strong protic methanol solvent effect plays a major role in the enhancement of luminescent properties of the strong electron-donating 4-methoxyphenyl and 4-methoxystyryl groups on the quinoline ring system for these complexes. A common interesting feature among the two series of complexes **4** and **5** is the observed lower emission intensity exhibited by the 2-(4-chlorophenyl)quinoline–based complexes **4c** and **5c**. The decrease in emission intensity may be due to the heavy atom effect of chlorine, which increases the probability of the intersystem crossing as the size of the molecule increases [42].

It is interesting to note that, under photoexcitation in the solid state, all of the Ir(III) complexes tuned the emission from the green to red light as shown (Figure 9 and Figure 10) compared to those in methanolic solution. All complexes emit at a common wavelength of λ_em_ = 697 nm with varied emission intensities. It has been reported that emission becomes more dominant in the solid state when more efficient energy transfer obtained from intermolecular energy transfer from the ligand to the complex are induced by shorter intermolecular distance in the solid state [41]. These emission profiles were envisioned to originate from the ligand-centred excited state, metal-centred excited state, and the MLCT excited state, since in the cyclometalated Ir complexes, the wave function of the excited triplet state, Φ_T_, responsible for phosphorescence is principally expressed to be:

Φ_T_ = a × Φ(LC)_T_ + b × Φ(MLCT)_T_, where a and b are the normalized coefficients and Φ(LC)_T_ and Φ(MLCT)_T_ are the wave functions of the ligand-centred (^3^π–π*) and the MLCT excited triplet state, respectively. Accordingly, the above equation showed that the excited triplet states of the iridium complexes are a mixture of Φ(LC)_T_ and Φ(MLCT)_T_, whereas, the triplet excited state is attributed to the dominantly ^3^π–π* excited state when a > b and the dominantly ^3^MLCT excited state when b > a [21,39,43,44,45,46,47,48,49]. The emission curves may be the product of transmission at longer wavelengths, which originated due to the interference from scattered light made possible through the higher-order transmission of monochromators. This would probably account for the observed reduction in emission intensities of the complexes **4a**–**d** and **5a**–**d** at higher excitation wavelengths 400 nm and 450 nm (not shown). The optical density of the samples due to sample preparation and concentration errors may also have contributed to the distortion of the emission spectra if the linear range of about 0.1 absorbance units is exceeded leading to intensity fluctuations as particles drift through the laser beam [50,51,52].

The phosphorescence spectra of the Ir complexes of **4a**–**d** shown in Figure 9 reveal the highest emission intensity for **4a** bearing a 2-phenyl ring. A slightly higher emission intensity was observed for the 2-(4-chlorophenyl)–substituted complex **4c** as compared to the analogous 2-(4-fluorophenyl)–substituted complex **4b**. The 2-(4-methoxyphenyl)–substituted complex **4d** was found to exhibit the lowest emission intensity than the other derivatives. Figure 10 shows the solid state phosphorescence emission spectra of complexes **5a**–**d**. The highest emission intensity was observed for complex **5c** with a 2-(4-chlorophenyl) and 4-(4-methoxystyryl) groups around the quinoline core. Within this series, complex **5b** with a 2-(4-fluorophenyl) group on the 4-methoxystyrylquinoline exhibits the lowest emission intensity than the other derivatives. Complexes **5a** and **5d** only showed moderate emission intensity. The presence of the 2-(4-fluorophenyl) substituent on the 4-(4-fluorostyryl)quinoline **4b** or 4-(4-methoxystyryl)quinoline framework of **5b** resulted in 50% reduction in emission intensity of these complexes. Although it has been reported that the characteristic red shift of the photoluminescent spectra in the solid state are thought to be related to the formation of intermolecular aggregation [41], these results demonstrate the influence of introducing electron-withdrawing groups on phenyl ring for the effective lowering of the HOMO energy level as electron releasing group raises the HOMO energy level in Ir(III) complexes for their phosphorescence properties. 

### 2.4. Electrochemical Studies

**Figure 11:**
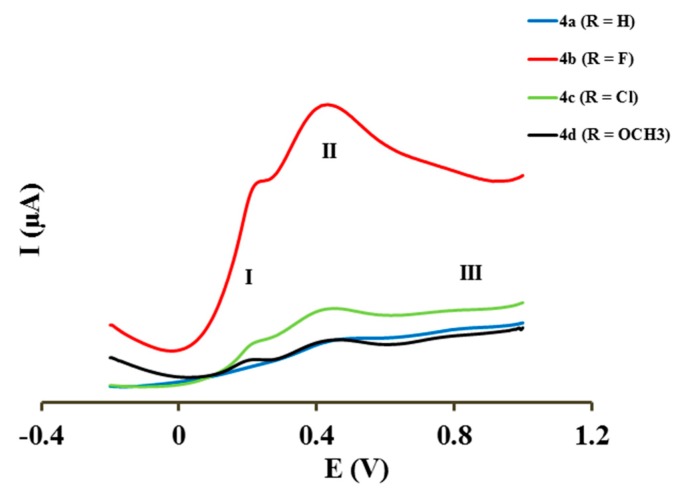
Square-wave voltammograms of (Ir(**4a**–**d**)_3_) complexes.

**Figure 12:**
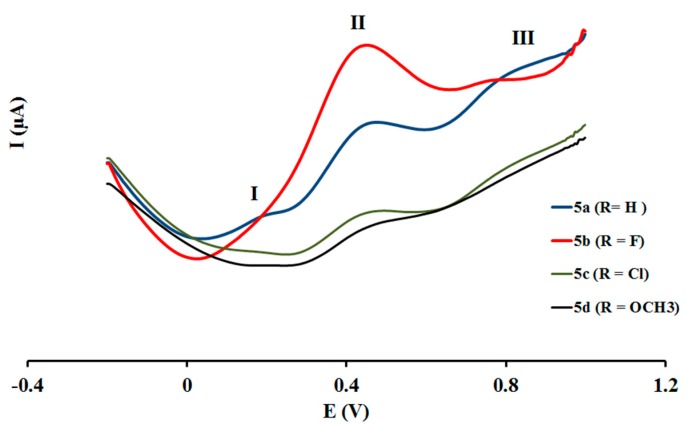
Square-wave voltammograms of (Ir(**5a**–**d**)_3_) complexes.

The electrochemical properties of the iridium metal complexes **4a**–**d** and **5a**–**d** in CHCl_3_ solution were investigated by cyclic voltammetry (refer to Figures S1 and S2 in the Supplementary Materials) and square-wave voltammetry (Figure 11 and Figure 12) using ferrocene/ferrocenium as the internal standard. All complexes show a similar profile with irreversible oxidation waves in the range 0.15–0.84 V and also undergo one electron reversible reduction profile (see Table S4 in the Supplementary Materials). The values of E_1/2_ at 50 to 60 mV s^−1^ scan rates are listed in Table S4 in the Supplementary Materials. Previous studies of iridium complexes assigned these oxidation peaks to both the metal-centered Ir^III^/Ir^IV^ oxidation process and the cyclometalated ligands [22,38,53,54,55]. The observed comparable oxidation potential values obtained for these complexes may be due to the same nature of the species involved in the process. In addition, upon switching on to cathodic sweep and electrode studied in the potential range +1.5 to −1.5 V and at a scan rate 50 mV s^−1^ (Table S4, Supplementary Materials), the voltammogram displays one reversible reduction peak with potentials range between −0.54 to −0.89 V, which were attributed to ligand-centered redox processes. Since all the complexes were differed only by nature of substituents and their positions either on the 2-phenyl- and/or 4-(4-phenylvinylene) rings, it is assumed that their HOMO and LUMO energy levels, and hence their redox potentials may be similar. Generally, compounds with electron withdrawing substituents were found to reduce more easily than those substituted with electron donating groups [56,57,58]. However, the reverse in reduction potentials was obtained for negative E_1/2_ values of the complexes **4b** (at −0.64 V) with a combination of moderately resonance donating 4-fluorophenyl and 4-fluorostyryl groups and complex **5d** (at −0.62 V) with only strongly electron-donating 4-methoxyphenyl and 4-methoxystyryl groups. This may possibly explain the contributory nature of the electron-donating group to reduction processes. Moreover, higher negative E_1/2_ values were obtained in complexes having a combination of an electron-donating and electron-withdrawing group on the same heterocyclic framework. Complex **4d** with a combination of 2-(4-methoxyphenyl) and 4-(4-fluorostyryl) groups was reduced at −0.82 V, whereas **5c** with a combination of 2-(4-chlorophenyl) and 4-(4-methoxystyryl) groups on the quinoline framework was reduced at −0.79 V. However, the reduction potentials in **4c** and **5c** at −0.54 V may further suggest that the electron acceptor performance of the ligand causes stabilization of the LUMO energy level of the complex [59]. In general, common trends observed in the electrochemistry are entirely closely related with those in the emission spectroscopy. It can reasonably, therefore, be confirmed that the oxidation potentials of these complexes primarily occur on the iridium metal centre, together with additional contributions from the cyclometalated quinoline framework. On the other hand, the reduction processes may be concentrated on the substituted styryl moiety of the quinoline chromophores. 

## 3. Materials and Methods

All chemical and reagents were analytically pure and used without further purification. Melting points were recorded on a Thermocouple digital melting point apparatus (Mettler Toledo LLC, Columbus, OH, USA) and are uncorrected. Microanalyses (C, H, and N) were carried out with a Fisons elemental analyzer (Fisons Thermo Scientific, Waltham, MA, USA) and Infrared spectra were recorded as powders using a Bruker VERTEX 70 FT-IR Spectrometer (Bruker Optics, Billerica, MA, USA) with a diamond ATR (attenuated total reflectance) accessory by using the thin-film method. The UV-Vis spectra were recorded on a Cecil CE 9500 (9000 Series) UV-Vis spectrometer (Cecil Instruments Cambridge, UK) while emission spectra were taken using a Perkin Elmer LS 45 fluorescence spectrometer (PerkinElmer, Llantrisant, UK) and Shimadzu RF-6000 Spectrofluoro-photometer (Shimadzu Europa GmbH, Duisburg, Germany). Quinine sulphate/1.0 N H_2_SO_4_ was used as a reference assuming a quantum yield of 0.55 with 360 nm excitation to determine luminescence quantum yields of the studied compounds, which was denoted as (Φf_x_) with the following equation: Φf_x_ = Φf_x_(F_s_A_x_/F_x_A_s_)(n_s_^2^/n_x_^2^), where the subscript *x* refers to the unknown sample and the subscript s to the standard, *A* is the absorbance at the excitation wavelength, *F* is the integrated emission across the band and *n* denotes the refractive index of the solvent [60]. For column chromatography, Merck Kieselgel 60 (0.063–0.200 mm) (Merck KGaA, Frankfurt, Germany) was used as stationary phase. NMR spectra were obtained as CDCl_3_ solutions using Varian Mercury 300 MHz NMR spectrometer (Varian Inc., Palo Alto, CA, USA) and the chemical shifts are quoted relative to the solvent peaks. Electrochemical experiment was performed using a PGSTAT 302 Autolab potentiostat (EcoChemie, Utrecht, The Netherlands) driven by the general purpose Electrochemical System data processing software (GPES, software version 4.9, Eco Chemie B.V, Kanaalweg, Utrecht, The Netherland). The electrochemical cell consists of a glassy carbon working electrode, platinum wire counter electrode and Ag/AgCl reference electrode. The oxidation and reduction measurements were recorded in a dichloromethane solution containing tetra(n-butyl)- ammonium tetrafluorophosphate as a supporting electrolyte at the scan rate of 50 mV s^−1^ under nitrogen conditions. Each oxidation potential was calibrated with the use of ferrocene/ferrocenium as a reference. The reported complexes were examined at a concentration of 10^−3^ M and supporting electrolyte at 10^−1^ M. The synthesis and analytical data of **1** have been described elsewhere [29]. 

### 3.1. Typical Procedure for the Suzuki–Miyaura Cross-Coupling of 1 with Arylboronic Acids

2-Aryl-4-chloroquinoline **1** (1 equiv.), 4-arylvinylboronic acid (1.2 equiv.), Pd(PPh_3_)_4_ (5% of 1), PCy_3_ (10% of 1) and K_2_CO_3_ (2.0 equiv.) in 4:1 dioxane-water (15 mL/mmol of 1) were added to a two-necked flask equipped with a stirrer bar, rubber septum and a condenser. The mixture was flushed for 20 min with argon gas and a balloon filled with argon gas was connected to the top of the condenser. The mixture was heated with stirring at 80–90 °C under argon atmosphere for 3 h and then allowed to cool to room temperature. The cooled mixture was poured into ice-cold water and the product was taken-up into chloroform. The combined organic extracts were washed with brine, dried over anhydrous MgSO_4_, filtered and then evaporated under reduced pressure. The residue was purified by column chromatography to afford **2**. The following products were prepared in this fashion:

4-(4-Fluorophenyvinyl)-2-phenylquinoline (**2a**). A mixture of **1a** (0.30 g, 1.25 mmol), 4-fluorophenyl vinylboronic acid (0.27 g, 1.63 mmol), Pd(PPh_3_)_4_ (0.07 g, 0.06 mmol), PCy_3_ (0.04 g, 0.13 mmol) and K_2_CO_3_ (0.35 g, 2.51 mmol) in aqueous dioxane (15 mL) afforded **2a** as a yellow solid (0.16 g, 42%), mp. 128–130 °C; R_f_ (chloroform) 0.52; ν_max_ (ATR) 481, 505, 672, 685, 754, 1228, 1508, 1578, 3056 cm^−1^; δ_H_ (CDCl_3_) 7.12 (t, 2H), 7.29 (d, *J* = 16.8 Hz, 1H), 7.48–7.59 (m, 6H), 7.70 (d, *J* = 16.8 Hz, 1H), 7.73 (d, *J* = 15 Hz, 1H), 7.99 (s, 1H), 8.13 (d, *J* = 7.5, 1H), 8.21 (d, *J* = 7.5 Hz, 3H); δ_C_ (75 MHz, CDCl_3_) 114.9, 115.8 (d, ^2^*J*_CF_ = 21.6 Hz), 123.0 (d, ^4^*J*_CF_ = 2.3 Hz), 123.1, 125.3, 126.2, 127.5, 128.6 (d, ^3^*J*_CF_ = 8.3 Hz), 128.8, 129.2, 129.5, 130.3, 132.7 (d, ^4^*J*_CF_ = 3.4 Hz), 133.5, 139.7, 143.3, 148.7, 157.1, 162.9 (d, ^1^*J*_CF_ = 247.4 Hz); Anal. Calcd. for C_23_H_16_NF: C, 84.89; H, 4.96; N, 4.31. Found: C, 84.94; H, 4.92; N, 4.38.

2-(4-Fluorophenyl)-4-(4-fluorophenyvinyl)quinoline (**2b**). A mixture of **1b** (0.30 g, 1.16 mmol), 4-fluorophenylvinylboronic acid (0.25 g, 1.51 mmol), Pd(PPh_3_)_4_ (0.07 g, 0.06 mmol), PCy_3_ (0.03 g, 0.12 mmol) and K_2_CO_3_ (0.32 g, 2.32 mmol) in aqueous dioxane (15 mL) afforded **2b** as a yellow solid (0.15 g, 38%), mp. 125–126 °C; Rf (chloroform) 0.72; ν_max_ (ATR) 481, 505, 629, 754, 837, 956, 1155, 1225, 1424, 1501, 1579, 3055, 2922 cm^−1^; δH (CDCl_3_): 7.13–7.22 (m, 4H), 7.34 (d, *J* = 15.3 Hz, 1H), 7.54–7.64 (m, 3H), 7.74 (d, *J* = 16.8 Hz, 1H), 7.76 (d, *J* = 16.8 Hz, 1H), 7.98 (s, 1H), 8.16–8.22 (m, 4H); δ_C_ (75 MHz, CDCl_3_) 114.7, 115.6, 115.7 (d, ^2^*J*_CF_ = 21.3 Hz), 115.8, 116.1, 118.4, 123.1 (d, ^4^*J*_CF_ = 2.6 Hz), 123.3, 124.5, 125.3, 126.3, 128.8 (d, ^3^*J*_CF_ = 8.0 Hz), 129.4 (d, ^3^*J*_CF_ = 8.6 Hz), 129.7, 130.3, 132.7, 132.8, 133.8, 135.9, 143.6, 148.7, 156.1, 161.7 (d, ^1^*J*_CF_ = 59.2 Hz), 165.0 (d, ^1^*J*_CF_ = 58.6 Hz); Anal. Calcd. for C_23_H_15_NF_2_: C, 80.44; H, 4.41; N, 4.08. Found: C, 80.70; H, 4.78; N, 4.25.

2-(4-Chlorophenyl)-4-(4-fluorophenyvinyl)quinoline (**2c**). A mixture of **1c** (0.09 g, 0.35 mmol), 4-fluorophenylvinylboronic acid (0.08 g, 0.46 mmol), Pd(PPh_3_)_4_ (0.02 g, 0.02 mmol), PCy_3_ (0.01 g, 0.04 mmol) and K_2_CO_3_ (0.09 g, 0.70 mmol) in aqueous dioxane (15 mL) afforded **2c** as off-white solid (0.15 g, 48%), mp. 98–101°C; *R*_f_ (chloroform) 0.43; ν_max_ (ATR) 459, 533, 689, 753, 826, 836, 1090, 1321, 1416, 1486, 1543, 1572, 1588, 2924 cm^−1^; δ_H_ (CDCl_3_) 7.01 (t, 1H), 7.36–7.41 (m, 1H), 7.49 (d, *J* = 9.0 Hz, 3H), 7.63 (t, 1H), 7.78 (t, 2H), 7.99 (s, 1H), 8.09 (d, *J* = 9.3 Hz, 3H), 8.15 (d, *J* = 7.5 Hz, 2H), 8.22 (d, *J* = 9.3 Hz, 1H); δ_C_ (75 MHz, CDCl_3_) 115.6 (d, ^2^*J*_CF_ = 21.6 Hz), 118.7, 123.9, 125.3, 127.5, 127.8 (d, ^3^*J*_CF_ = 7.7 Hz), 128.7, 129.1, 130.0, 130.8, 131.5, 133.4 (d, ^4^*J*_CF_ = 3.4 Hz), 136.1, 136.9, 143.4, 148.9, 155.9, 162.3 (d, ^1^*J*_CF_ = 245.9 Hz); Anal. Calcd. for C_23_H_15_NFCl: C, 76.86; H, 4.21; N, 3.90. Found: C, 76.42; H, 4.54; N, 3.55.

4-(4-Fluorophenyvinyl)-2-(4-methoxyphenyl)quinoline (**2d**). A mixture of **1d** (0.30 g, 1.11 mmol), 4-fluorophenylvinylboronic acid (0.24 g, 1.44 mmol), Pd(PPh_3_)_4_ (0.07 g, 0.06 mmol), PCy_3_ (0.03 g, 0.11 mmol) and K_2_CO_3_ (0.31 g, 2.22 mmol) in aqueous dioxane (15 mL) afforded **2d** as a brilliant yellow solid (0.19 g, 48%), mp. 125–128 °C; *R*_f_ (chloroform) 0.50; ν_max_ (ATR) 481, 753, 838, 1091, 1173, 1229, 1416, 1486, 1503, 1543, 1586, 2927, 3045 cm^−1^; δ_H_ (CDCl_3_) 3.88 (s, 3H), 6.96 (m, 1H), 7.04–7.15 (m, 3H), 7.32 (d, *J* = 16.8 Hz, 1H), 7.52 (t, 1H), 7.58–7.62 (m, 2H), 7.71 (d, *J* = 15.3 Hz, 1H), 7.73 (d, *J* = 16.8 Hz, 1H), 7.97 (s, 1H), 8.12–8.18 (m, 3H); δ_C_ (75 MHz, CDCl_3_) 55.4, 114.2, 114.7, 115.9 (d, ^2^*J*_CF_ = 21.6 Hz), 118.3, 123.2, 123.3 (d, ^4^*J*_CF_ = 2.3 Hz), 124.4, 125.1, 125.9, 128.7 (d, ^3^*J*_CF_ = 8.0 Hz), 128.8, 129.5, 129.7, 130.2, 130.7 (d, ^3^*J*_CF_ = 8.0 Hz), 132.3, 132.8 (d, ^4^*J*_CF_ = 3.4 Hz), 133.5, 143.3, 148.7, 156.7, 160.8, 162.9 (d, ^1^*J*_CF_ = 247.4 Hz); Anal. Calcd. for C_24_H_18_NFO: C, 81.10; H, 5.11; N, 3.94. Found: C, 81.43; H, 5.39; N, 4.12.

4-(4-Methoxyphenyvinyl)-2-phenylquinoline (**3a**). A mixture of **1a** (0.30 g, 1.25 mmol), 4-methoxyphenylvinylboronic acid (0.27 g, 1.50 mmol), Pd(PPh_3_)_4_ (0.07 g, 0.06 mmol), PCy_3_ (0.04 g, 0.12 mmol) and K_2_CO_3_ (0.35 g, 2.51 mmol) in aqueous dioxane (15 mL) afforded **3a** as a yellow oil (0.20 g, 67%); *R*_f_ (chloroform) 0.44; ν_max_ (ATR) 515, 691, 754, 767, 1028, 1172, 1250, 1508, 1583, 1604, 2834, 2930 cm^−1^; δ_H_ (CDCl_3_) 3.86 (s, 3H), 6.97 (d, *J* = 9.0 Hz, 2H), 7.36 (d, *J* = 15.9 Hz, 1H), 7.49–7.61 (m, 6H), 7.71 (d, *J* = 15.3 Hz, 1H), 7.74 (d, *J* = 15.2 Hz, 1H), 8.04 (s, 1H), 8.18–8.23 (m, 4H); δ_C_ (75 MHz, CDCl_3_) 55.3, 113.7, 114.3, 114.7, 118.8, 120.9, 123.3, 123.7, 124.7, 125.4, 126.1, 126.2, 127.4, 127.5, 128.5, 128.7, 128.8, 129.2, 129.3, 129.4, 129.7, 130.1, 130.2, 130.4, 133.6, 134.5, 139.8, 143.9, 148.7, 157.1, 160.1; Anal. Calcd. for C_24_H_19_NO: C, 85.42; H, 5.68; N, 4.15. Found: C, 85.77; H, 5.85; N, 4.34. 

2-(4-Fluorophenyl)-4-(4-methoxyphenylvinyl)quinoline (**3b**). A mixture of **1b** (0.30 g, 1.16 mmol), 4-methoxyphenylvinylboronic acid (0.25 g, 1.39 mmol), Pd(PPh_3_)_4_ (0.07 g, 0.06 mmol), PCy_3_ (0.03 g, 0.12 mmol) and K_2_CO_3_ (0.32 g, 2.32 mmol) in aqueous dioxane (15 mL) afforded 3b as a light yellow solid (0.09 g, 42%), mp. 95–98 °C; *R*_f_ (chloroform) 0.67; ν_max_ (ATR) 755, 839, 964, 1156, 1172, 1250, 1416, 1486, 1503, 1544, 1582, 2958 cm^−1^; δ_H_ (CDCl_3_) 3.87 (s, 3H), 6.97 (d, *J* = 8.1 Hz, 2H), 7.22 (t, 3H), 7.36 (d, *J* = 15.9 Hz, 1H), 7.57 (m, 4H), 7.71 (d, *J* = 15.3 Hz, 1H), 7.99 (s, 1H), 8.15–8.21 (m, 3H); δ_C_ (75 MHz, CDCl_3_) 55.4, 113.7, 114.1, 114.3, 114.4, 115.6 (d, ^4^*J*_CF_ = 5.1 Hz), 115.9 (d, ^4^*J*_CF_ = 5.2 Hz), 118.5, 120.9, 123.5 (d, ^2^*J*_CF_ = 22.8 Hz), 124.7, 126.2, 126.3, 128.5, 129.3, 129.4 (d, ^3^*J*_CF_ = 8.0 Hz), 129.6, 129.8, 130.2, 130.1, 130.2, 130.4, 133.8, 134.6, 160.2 (d, ^1^*J*_CF_ = 247.4 Hz); Anal. Calcd. for C_24_H_18_NOF: C, 81.10; H, 5.11; N, 3.94. Found: C, 81.40; H, 5.36; N, 4.16.

2-(4-Chlorophenyl)-4-(4-methoxyphenylvinyl)quinoline (**3c**). A mixture of **1c** (0.25 g, 0.91 mmol), 4-methoxyphenylvinylboronic acid (0.20 g, 1.09 mmol), Pd(PPh_3_)_4_ (0.05 g, 0.05 mmol), PCy_3_ (0.03 g, 0.09 mmol) and K_2_CO_3_ (0.25 g, 1.82 mmol) in aqueous dioxane (15 mL) afforded **3c** as a bright yellow solid (0.11 g, 41%), mp. 144–146 °C; *R*_f_ (chloroform) 0.70; ν_max_ (ATR) 518, 529, 763, 822, 835, 966, 1027, 1172, 1253, 1509, 1582 cm^−1^; δ_H_ (CDCl_3_) 3.87 (s, 3H), 6.96 (d, *J* = 8.4 Hz, 2H), 7.35 (d, *J* = 15.9 Hz, 1H), 7.51 (d, *J* = 8.4 Hz, 2H), 7.58–7.70 (m, 4H), 7.70 (d, *J* = 16.4 Hz, 2H), 7.99 (s, 1H), 8.14–8.20 (m, 3H); δ_C_ (75 MHz, CDCl_3_) 55.4, 114.3, 120.8, 123.3, 125.4, 126.3, 127.4, 128.5, 128.8, 128.9, 129.3, 129.6, 130.3, 133.8, 134.6, 135.4, 138.3, 144.1, 148.7, 155.8, 160.2; Anal. Calcd. for C_24_H_18_NOCl: C, 77.61; H, 4.89; N, 3.77. Found: C, 78.02; H, 4.76; N, 3.58

2-(4-Methoxyphenyl)-4-(4-methoxyphenylvinyl)quinoline (**3d**). A mixture of **1d** (0.30 g, 1.11 mmol), 4-methoxyphenylvinylboronic acid (0.24 g, 1.33 mmol), Pd(PPh_3_)_4_ (0.07 g, 0.06 mmol), PCy_3_ (0.03 g, 0.11 mmol) and K_2_CO_3_ (0.31 g, 2.22 mmol) in aqueous dioxane (15 mL) afforded **3d** as a cream-white solid (0.19 g, 47%), mp. 75–78 °C; *R*_f_ (chloroform) 0.22; ν_max_ (ATR) 533, 751, 823, 1026, 1171, 1250, 1326, 1491, 1574, 2922 cm^−1^; δ_H_ (CDCl_3_) 3.88 (s, 6H), 7.04 (d, *J* = 9.3 Hz, 3H), 7.58 (t, 2H), 7.75 (t, 2H), 7.92 (s, 1H), 8.11 (d, *J* = 7.8 Hz, 2H), 8.18 (t, 5H); δ_C_ (75 MHz, CDCl_3_) 55.4, 114.1, 114.3, 118.6, 123.9, 124.9, 126.8, 127.4, 127.5, 128.8, 129.8, 130.4, 131.0, 131.2, 142.9, 149.0, 156.8, 161.1; Anal. Calcd. for C_25_H_21_NO_2_: C, 81.71; H, 5.76; N, 3.81. Found: C, 82.05; H, 5.98; N, 3.89.

### 3.2. Synthetic Method and Characterization of Iridium(III) Complexes ***4a**–**d*** and ***5a**–**d***

The reported cyclometalated Ir(III) complexes **4a**–**d**) and **5a**–**d** were synthesized by a slight modification to the method as previously reported in the literature [35]. In a 2-necked round bottomed flask, each of the ligand **2a**–**d** or **3a**–**d** (1 equiv.) was dissolved in 3:1 2-ethoxyethanol–water (*v/v*), and the mixture was purged with argon gas for 5 min, before iridium trichloride hydrate [IrCl_3_(H_2_O)_3_] (one-third equimolar ratio of ligand) was added and the mixture heated at 120 °C under argon atmosphere for 12 h. After reaction, the solution was allowed to cool to room temperature and then filtered. The filtrate was concentrated under vacuum to remove 2-ethoxyethanol, and the crude complex product was further purified by column chromatography in ethyl acetate-toluene (9:1) to obtain brown powder. The iridium complexes were characterized by ^1^H NMR and elemental analysis. The ^1^H NMR peaks of the complexes are very similar to those of the starting ligands due to chemical equivalence. The principal difference is the downfield shift due to the loss of one hydrogen atom and coordination of the N^˄^C-ligand to the iridium metal center.

Complex **4a**: Brown solid (54 mg, 27%), ^1^H NMR (300 MHz, CDCl_3_): δ 6.91 (t), 7.02 (d, *J* = 7.5 Hz), 7.07 (d, *J* = 7.5 Hz), 7.12 (s), 7.19 (t), 7.28 (d, *J* = 7.8 Hz), 7.38 (d, *J* = 7.5 Hz), 7.44 (d, *J* = 6.0 Hz), 7.54–7.99 (m), 8.05 (d, *J* = 3.3 Hz), 8.28 (d, *J* = 7.8), 8.50 (d, *J* = 7.5 Hz). Anal. Calcd. for IrC_69_H_45_N_3_F_3_: C, 71.12; H, 3.89; N, 3.61. Found: C, 71.45; H, 3.96; N, 3.87. 

Complex **4b**: Brown solid (62 mg, 30%), ^1^H NMR (300 MHz, CDCl_3_): δ 6.98 (s), 7.11 (s), 7.14–7.23 (m), 7.44 (d, *J* = 15.3 Hz), 7.07 (d, *J* = 7.5 Hz), 7.12 (s), 7.19 (t), 7.28 (d, *J* = 7.8 Hz), 7.38 (d, *J* = 7.5 Hz), 7.44 (d, *J* = 6.0 Hz), 7.54–7.67 (m), 7.73 (s), 7.77–7.81 (m), 8.00 (s), 8.20 (d, *J* = 7.5 Hz), 8.40 (s, br). Anal. Calcd. for IrC_69_H_42_N_3_F_6_: C, 67.97; H, 3.47; N, 3.45. Found: C, 67.83; H, 3.56; N, 3.54.

Complex **4c**: Pink solid. Yield: 25% (31 mg), ^1^H NMR (300 MHz, CDCl_3_): δ 6.61 (d, *J* = 12.3 Hz), 6.81 (d, *J* = 3.0 Hz), 6.86 (d, *J* = 3.0 Hz), 6.99–7.07 (m), 7.37–7.41 (m), 7.50 (d, *J* = 6.0 Hz), 7.64 (t), 7.80 (t), 7.94 (s), 8.11 (d, *J* = 7.8 Hz), 8.22 (t). Anal. Calcd. for IrC_69_H_42_N_3_F_3_Cl_3_: C, 65.32; H, 3.34; N, 3.31. Found: C, 65.53; H, 3.48; N, 3.47.

Complex **4d**: Dark brown solid (30 mg, 28%), ^1^H NMR (300 MHz, CDCl_3_): δ 3.65 (s, OCH_3_), 6.80 (d, *J* = 7.8 Hz), 6.97 (d, *J* = 7.8 Hz), 7.09 (t), 7.17 (d, *J* = 7.8 Hz), 7.52–7.68 (m), 7.73–7.79 (m), 7.84 (d, *J* = 9.0 Hz), 7.96 (d, *J* = 7.5 Hz), 8.00 (s), 8.13 (d, *J* = 9.3 Hz), 8.24 (d, *J* = 9.3 Hz), 8.41 (br, s), 8.64 (s). Anal. Calcd. for IrC_72_H_51_N_3_F_3_O_3_: C, 72.16; H, 4.29; N, 3.51. Found: C, 72.42; H, 4.27; N, 3.66.

Complex **5a**: Brown solid (74 mg, 53%), ^1^H NMR (300 MHz, CDCl_3_): δ 3.94 (s, OCH_3_), 7.16 (d, *J* = 9.3 Hz), 7.30 (t), 7.43 (t), 7.62 (d, *J* = 9.3 Hz), 7.71 (m), 7.87 (s), 7.93 (d, *J* = 9.3 Hz), 8.14 (d, *J* = 7.5 Hz), 8.34 (br, s). Anal. Calcd. for IrC_72_H_54_N_3_O_3_: C, 71.98; H, 4.53; N, 3.50. Found: C, 71.85; H, 4.33; N, 3.57.

Complex **5b**: Brown solid (79 mg, 57%), ^1^H NMR (300 MHz, CDCl_3_): δ 3.74 (s, OCH_3_), 6.93 (d, *J* = 7.8 Hz), 7.60 (d, *J* = 3.0 Hz), 7.61 (d, *J* = 13.5 Hz), 7.67 (d, *J* = 7.5 Hz), 7.80 (s), 7.95 (d, *J* = 7.5 Hz), 8.02 (d, *J* = 7.5 Hz), 8.53 (br, s). Anal. Calcd. for IrC_72_H_51_N_3_F_3_O_3_: C, 68.88; H, 4.09; N, 3.35. Found: C, 68.62; H, 4.37; N, 3.53.

Complex **5c**: Brown solid (74 mg, 42%), ^1^H NMR (300 MHz, CDCl_3_): δ 3.96 (s, OCH_3_), 7.14 (d, *J* = 8.7 Hz), 7.56 (d, *J* = 8.7 Hz), 7.52 (d, *J* = 8.7 Hz), 7.77–7.80 (br, s), 8.02 (d, *J* = 8.1 Hz), 8.16 (d, *J* = 8.4 Hz), 8.31 (d, *J* = 8.4 Hz). Anal. Calcd. for IrC_72_H_51_N_3_Cl_3_O_3_: C, 66.28; H, 3.94; N, 3.22. Found: C, 66.12; H, 3.80; N, 3.54.

Complex **5d**: Dark brown solid (85 mg, 45%), ^1^H NMR (300 MHz, CDCl_3_): δ 3.68 (s, OCH_3_), 3.91 (s, OCH_3_), 6.88 (d, *J* = 7.8 Hz), 7.11 (d, *J* = 9.3 Hz), 7.56 (d, *J* = 7.5 Hz), 7.61 (s), 7.76 (s), 7.82 (t), 8.04 (t), 8.46 (d, *J* = 9.0 Hz). Anal. Calcd. for IrC_75_H_60_N_3_O_6_: C, 69.75; H, 4.68; N, 3.25. Found: C, 69.52; H, 4.87; N, 3.64.

### 3.3. DFT Computation

The ligand (**2a**) and its corresponding iridium(III) complex (**4a**) were optimised in the gas phase using the DFT method. The B3LYP functional was used in conjunction with the 6-31G(d,p) basis set for all atoms except for iridium, where the LANL2DZ ECP basis set was used. The ligand was also optimised in three solvents, namely methanol, chloroform and dimethyl formamide. The complex was optimised in methanol. Solvent computations were based on the PCM model [61,62]. Frequency computations were performed on all of the optimised structures to confirm the nature of the stationary points. All the structures were optimised as ground states without any imaginary frequency. To simulate the UV-Vis spectra, Time-dependent density functional theory (TDDFT) computations were performed for the ligand in chloroform and the complex in methanol. All computations were carried out using Gaussian 09 software running on Gridchem [63,64,65].

## 4. Conclusions

This study has shown that the electronic absorption, emission and electrochemical properties of the complexes originate from the ligands and the both electron-donating and electron-withdrawing groups on the quinoline framework play an important role in the determination of the optical properties, the quantum yields and the redox properties. It has been shown among the d_6_ metals, that the cyclometalated iridium(III) complexes represent the most promising high performance emitters due to their strong Ir-C bonds, which ensure good photo- and thermal stability, thermally accessible destabilization and non-emissive metal centred (MC) states. The molecular framework of the quinoline ligands used in this study is linked directly to the aryl ring or through a pi-conjugated bridge leading to low redox potentials and a greater tendency to undergo electron transfer reactions. It is quite interesting to observe the tunable photoluminescence from blue to green to red for the iridium(III) complexes on the basis of π→π* elongation of conjugation, electron-withdrawing or electron-donating substituent at the 4-position of the phenylvinylene moiety of the quinoline framework. The reported Ir(III) complexes may find use as potential emissive dopants, fluorescent marker, electron transport materials for OLEDs application, and drug discovery.

## Figures and Tables

**Table 1:** Selected bond lengths (Ir–N and Ir–C) in Å of complex **4a** and their comparison with literature values.

	4a	Literature [30]
Ir–N	2.299	2.173
Ir–C	2.018	2.017

## Supplementary Materials

The following are available online at www.mdpi.com/1996-1944/10/10/1061/s1, Figure S1: Cyclic voltammograms of [Ir(**4a**–**d**)_3_] showing the quasi-reversible oxidation potential against ferrocene/ferrocenium; Figure S2: Cyclic voltammograms of [Ir(**4a**–**d**)_3_] showing the reversible reduction potential against ferrocene/ferrocenium; Figure S3: Cyclic voltammograms of [Ir(**5a**-**d**)_3_] showing the quasi-reversible oxidation potential against ferrocene/ferrocenium; Figure S4: Cyclic voltammograms of [Ir(**5a**–**d**)_3_] showing the reversible reduction potential against ferrocene/ferrocenium; Table S1: Cartesian coordinates of ligand 2a in the gas phase; Table S2: Cartesian coordinates of complex 4a in the gas phase; Table S3: Summary of Physico-chemical properties of ligands (**2a**–**d**) and (**3a**–**d**) and complexes (**4a**–**d**) and (**5a**–**d**); Table S4: Summary of photophysical data of ligands (**2a**–**d**) and (**3a**–**d**); Table S5: Summary of photophysical and electrochemical data of [Ir(**4a**–**d**)_3_] and [Ir(**5a**–**d**)_3_].
