# Supplementary material for: Spectroscopic, Electrochemical and DFT Studies of Phosphorescent Homoleptic Cyclometalated Iridium(III) Complexes Based on Substituted 4-Fluorophenylvinyl- and 4-Methoxyphenylvinylquinolines
Source: Materials (Basel). 2017 Sep 21;10(10):1061. doi: 10.3390/ma10101061 (PMC5666912; doi:10.3390/ma10101061)

Table S1: Cartesian coordinates of ligand **2a** in the gas phase.

7	2.957240000	1.117782000	-0.134224000
6	1.980176000	2.059920000	-0.047978000
6	2.629438000	-0.158060000	0.016569000
6	2.375778000	3.419717000	-0.171180000
6	0.596265000	1.749150000	0.168986000
6	1.286060000	-0.562339000	0.235892000
6	3.736160000	-1.152066000	-0.051175000
6	1.456854000	4.435159000	-0.046906000
1	3.427566000	3.615309000	-0.351346000
6	-0.316567000	2.824847000	0.330677000
6	0.250145000	0.357016000	0.267589000
1	1.055466000	-1.614066000	0.364591000
6	3.505389000	-2.511916000	-0.314604000
6	0.102032000	4.133881000	0.224423000
1	1.771623000	5.470740000	-0.136385000
1	-1.349809000	2.612033000	0.575211000
6	-1.117291000	-0.165330000	0.402476000
6	4.563723000	-3.418156000	-0.367861000
1	2.498872000	-2.868833000	-0.507422000
6	6.115387000	-1.630834000	0.096538000
1	-0.612004000	4.940444000	0.362068000
1	-1.186683000	-1.092118000	0.969876000
6	-2.230334000	0.313107000	-0.190240000
6	5.872460000	-2.982732000	-0.158419000
1	4.364467000	-4.464530000	-0.580412000
1	7.131368000	-1.282287000	0.258847000
1	-2.141360000	1.178602000	-0.842235000
6	-3.580026000	-0.253786000	-0.102429000
1	6.696311000	-3.689408000	-0.197690000
6	-3.949238000	-1.230297000	0.843758000
6	-4.565865000	0.197684000	-0.999701000
6	-5.238625000	-1.748569000	0.880561000
1	-3.224701000	-1.579633000	1.572133000
6	-5.861994000	-0.310506000	-0.977237000
1	-4.306631000	0.956043000	-1.733334000
6	-6.177520000	-1.281622000	-0.035273000
1	-5.529566000	-2.497314000	1.609188000
1	-6.621561000	0.032662000	-1.670900000

9	-7.429582000	-1.780674000	0.000677000
6	5.060062000	-0.724826000	0.146236000
1	5.237384000	0.327306000	0.337062000

Table S2: Cartesian coordinates of complex **4a** in the gas phase.

77	0.167380000	-0.248842000	1.001674000
6	0.348848000	2.479590000	-0.936039000
6	-1.300013000	2.407775000	0.728054000
6	1.323663000	1.806167000	-1.713177000
6	0.062789000	3.850158000	-1.238382000
6	-1.602749000	3.775108000	0.494755000
6	1.990175000	2.449545000	-2.734363000
1	1.524144000	0.770141000	-1.486172000
6	0.761178000	4.478193000	-2.299607000
6	-0.944938000	4.518531000	-0.461566000
1	-2.326448000	4.262351000	1.134629000
6	1.711035000	3.798807000	-3.032913000
1	2.729640000	1.907278000	-3.316530000
1	0.525814000	5.505907000	-2.553547000
1	2.232671000	4.297477000	-3.844322000
6	-1.994404000	1.617668000	1.747832000
6	-3.146264000	2.095945000	2.404381000
6	-1.447008000	0.341284000	2.058452000
6	-3.765553000	1.340641000	3.389894000
1	-3.575004000	3.056742000	2.135751000
6	-2.095504000	-0.390464000	3.073869000
6	-3.226385000	0.094264000	3.727864000
1	-4.654915000	1.714215000	3.889002000
1	-1.703108000	-1.358438000	3.364190000
1	-3.693841000	-0.503924000	4.506773000
7	-0.319174000	1.787191000	0.050669000
6	-1.238966000	5.943243000	-0.655769000
1	-0.407082000	6.566136000	-0.973601000
6	-2.450180000	6.507803000	-0.472009000
1	-3.287263000	5.856027000	-0.225743000
6	-2.793430000	7.926250000	-0.602268000
6	-1.830021000	8.946670000	-0.733996000
6	-4.150356000	8.301045000	-0.589306000
6	-2.203534000	10.279428000	-0.863445000
1	-0.773355000	8.699546000	-0.720512000
6	-4.542102000	9.631342000	-0.716655000
1	-4.910889000	7.532396000	-0.481752000
6	-3.558256000	10.602913000	-0.854747000

1	-1.466623000	11.069318000	-0.960971000
1	-5.587028000	9.921606000	-0.709039000
7	2.170998000	-0.801653000	0.020249000
6	2.435342000	-1.685423000	-1.003775000
6	3.201247000	-0.273169000	0.703190000
6	1.357254000	-2.199054000	-1.765437000
6	3.768075000	-2.075084000	-1.356223000
6	4.539815000	-0.654010000	0.423726000
6	2.865497000	0.669762000	1.772683000
6	1.576890000	-3.052084000	-2.826023000
1	0.358199000	-1.898176000	-1.488976000
6	3.958093000	-2.945584000	-2.458258000
6	4.855636000	-1.540464000	-0.583387000
1	5.329441000	-0.281150000	1.062252000
6	3.855071000	1.404085000	2.457477000
6	1.487980000	0.805584000	2.104062000
6	2.888051000	-3.430651000	-3.180275000
1	0.731738000	-3.428049000	-3.395334000
1	4.966378000	-3.217047000	-2.751143000
6	6.244209000	-1.945032000	-0.833320000
6	3.511952000	2.264616000	3.490188000
1	4.900112000	1.318194000	2.175523000
6	1.180213000	1.678043000	3.167644000
1	3.055515000	-4.094172000	-4.023393000
1	6.392514000	-2.955820000	-1.203713000
6	7.317388000	-1.151244000	-0.638474000
6	2.164943000	2.391023000	3.848126000
1	4.279759000	2.829570000	4.010695000
1	0.147672000	1.797392000	3.474997000
1	7.142282000	-0.119358000	-0.337234000
6	8.726815000	-1.506132000	-0.823005000
1	1.881961000	3.052425000	4.663919000
6	9.172160000	-2.827455000	-1.029355000
6	9.694480000	-0.484124000	-0.788911000
6	10.520254000	-3.114491000	-1.210701000
1	8.458792000	-3.645275000	-1.034363000
6	11.048902000	-0.754025000	-0.967616000
1	9.375601000	0.541497000	-0.623893000
6	11.441319000	-2.070185000	-1.179256000
1	10.869142000	-4.129680000	-1.366026000

1	11.795423000	0.032313000	-0.943883000
7	-1.337146000	-1.644359000	-0.035212000
6	-2.223342000	-1.383660000	-1.057326000
6	-1.434952000	-2.809725000	0.626486000
6	-2.031765000	-0.222367000	-1.845697000
6	-3.300889000	-2.270215000	-1.389818000
6	-2.497566000	-3.711092000	0.361687000
6	-0.427419000	-3.064483000	1.658608000
6	-2.841764000	0.046379000	-2.928187000
1	-1.218143000	0.436169000	-1.581514000
6	-4.095153000	-1.976174000	-2.526883000
6	-3.472537000	-3.446970000	-0.578576000
1	-2.589019000	-4.600168000	0.973674000
6	-0.300545000	-4.322033000	2.282349000
6	0.421448000	-1.978054000	2.011901000
6	-3.875965000	-0.843169000	-3.282537000
1	-2.665027000	0.938360000	-3.522239000
1	-4.861768000	-2.680369000	-2.825086000
6	-4.604659000	-4.377853000	-0.667346000
6	0.649704000	-4.528431000	3.272164000
1	-0.933340000	-5.152462000	1.983168000
6	1.367047000	-2.223438000	3.027702000
1	-4.488688000	-0.647123000	-4.157415000
1	-4.362516000	-5.401965000	-0.387802000
6	-5.891097000	-4.065675000	-0.930765000
6	1.480628000	-3.466388000	3.647166000
1	0.745774000	-5.501381000	3.745194000
1	2.025526000	-1.422820000	3.345010000
1	-6.146770000	-3.023717000	-1.107620000
6	-7.029834000	-4.988867000	-0.950072000
1	2.223545000	-3.611244000	4.428287000
6	-6.891325000	-6.391317000	-0.933382000
6	-8.332785000	-4.457390000	-0.994820000
6	-8.002896000	-7.226299000	-0.943558000
1	-5.902425000	-6.838283000	-0.926430000
6	-9.457044000	-5.279148000	-1.005269000
1	-8.464075000	-3.378879000	-1.014346000
6	-9.273178000	-6.656238000	-0.977017000
1	-7.902496000	-8.306260000	-0.934205000
1	-10.461517000	-4.871339000	-1.035531000

9	12.749077000	-2.347163000	-1.351219000
9	-3.922120000	11.894933000	-0.976522000
9	-10.351809000	-7.464502000	-0.990483000

Table S3: Summary of Physico-chemical properties of ligands (**2a-d**) and (**3a-d**) and complexes (**4a-d**) and (**5a-d**).

Compd.	R	R'	% Yield	Colour
**2a**	4-H	-F	42	Yellow solid
**2b**	4-F	-F	38	Yellow solid
**2c**	4-Cl	-F	48	Yellow solid
**2d**	4-OCH_3	-F	48	Brilliant Yellow solid
**3a**	4-H	-OCH_3	67	Yellow Oil
**3b**	4-F	-OCH_3	42	Brilliant Yellow solid
**3c**	4-Cl	-OCH_3	41	White solid
**3d**	4-OCH_3	-OCH_3	47	Bright Yellow solid
**4a**	4-H	-F	27	Brown solid
**4b**	4-F	-F	30	Brown Solid
**4c**	4-Cl	-F	25	Pink Solid
**4d**	4-OCH_3	-F	28	Dark Brown solid
**5a**	4-H	-OCH_3	53	Brown solid
**5b**	4-F	-OCH_3	57	Brown solid
**5c**	4-Cl	-OCH_3	42	Brown solid
**5d**	4-OCH_3	-OCH_3	45	Dark Brown solid

Table S4: Summary of photophysical data of ligands (**2a-d**) and (**3a-d**).

	Abs. λ_{max} (nm)[a]	(ε) × 10^4 Mol^{-1} cm^{-1}	λ_{em} (nm)[b] $CHCl_3$	λ_{em} (nm) DMF	λ_{em} (nm) CH_3OH	Quantum Yield (Φ)[c] $CHCl_3$	Quantum Yield (Φ) DMF	Quantum Yield (Φ) CH_3OH	Stokes Shift [d] ($CHCl_3$)
**2a**	265, 335	0.42, 0.29	449	409	449	0.23	0.11	0.23	184, 114
**2b**	278, 337	0.78, 0.25	454	411	439	0.27	0.14	0.27	176, 117
**2c**	267, 328	0.66, 0.25	454	406	462	0.30	0.27	0.30	187, 126
**2d**	293, 338	0.57, 0.45	478	442	482	0.16	0.15	0.16	185, 140
**3a**	261, 340	0.52, 0.24	465	452	450	0.31	0.14	0.30	204, 125
**3b**	262, 354	0.28, 0.17	470	438	480	0.45	0.14	0.23	208, 116
**3c**	266, 348	0.43, 0.35	460	454	440	0.23	0.06	0.47	193, 111
**3d**	278, 338	0.51, 0.19	455	389	470	0.40	0.35	0.25	177, 117

[a] Absorption spectra, [b]emission spectra, [c]luminescence quantum yields (Φ) and [d]Stokes-shifts were measured relative to chloroform solutions at 298 K. Wavelengths of excitation light are in the range of 350 – 385 nm.

Table S5: Summary of photophysical and electrochemical data of [Ir(**4a-d**)$_3$] and [Ir(**5a-d**)$_3$].

	Abs. λ_{max} (nm)[a]	(ε) × 10^4 Mol^{-1} cm^{-1}	λ_{em} (nm)[b] CH_3OH	Quantum Yield (Φ)[c] CH_3OH	E_{ox} (V)[d]	E_{re} (V)[e]
**4a**	258, 335, 396	1.65, 0.55, 0.13	499	0.59	0.41, 0.81	–0. 63
**4b**	260, 329, 392	1.90, 0.70, 0.11	530	0.47	0.22, 0.41, 0.76	–0.64
**4c**	265, 342	2.17, 0.43	489	0.70	0.21, 0.42, 0.76	–0.54, –0.73
**4d**	277, 340, 398	2.43, 0.62, 0.23	470	0.56	0.20, 0.41, 0.81	–0.67, –0.82
**5a**	258, 275, 336	0.71, 053, 0.27	500	0.62	0.17, 0.45, 0.77	–0.89
**5b**	260, 280, 324	1.53, 1.07, 0.60	530	0.57	0.42, 0.75	–0.57
**5c**	264, 324, 381	1.71, 0.62, 0.16	489	0.50	0.15, 0.42, 0.80	– 0.54, – 0.79
**5d**	284, 349, 380	1.11, 0.55, 0.61	545	0.58	0.20, 0.43, 0.78	–0.62

[a] Absorption spectra, [b]emission spectra, [c]luminescence quantum yields (Φ) were measured in methanol solutions at 298 K. Wavelengths of excitation light are in the range of 350 – 450 nm. [d&e]Electrochemical data versus Fc^+/Fc (Fc is Ferrocene) as internal standard.

Fig. S1: Cyclic voltammograms of [Ir(**4a-d**)$_3$] showing the quasi-reversible oxidation potential against ferrocene/ferrocenium.

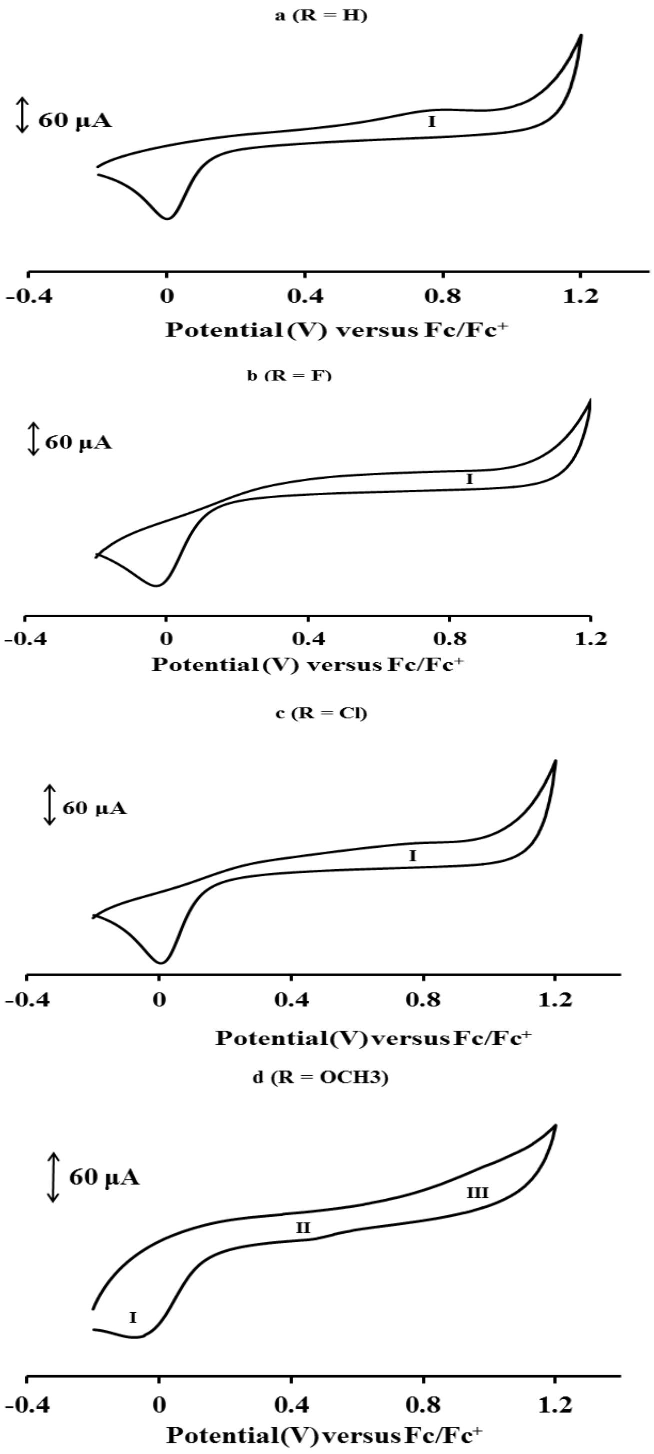

Fig. S2: Cyclic voltammograms of [Ir(**4a-d**)$_3$] showing the reversible reduction potential against ferrocene/ferrocenium.

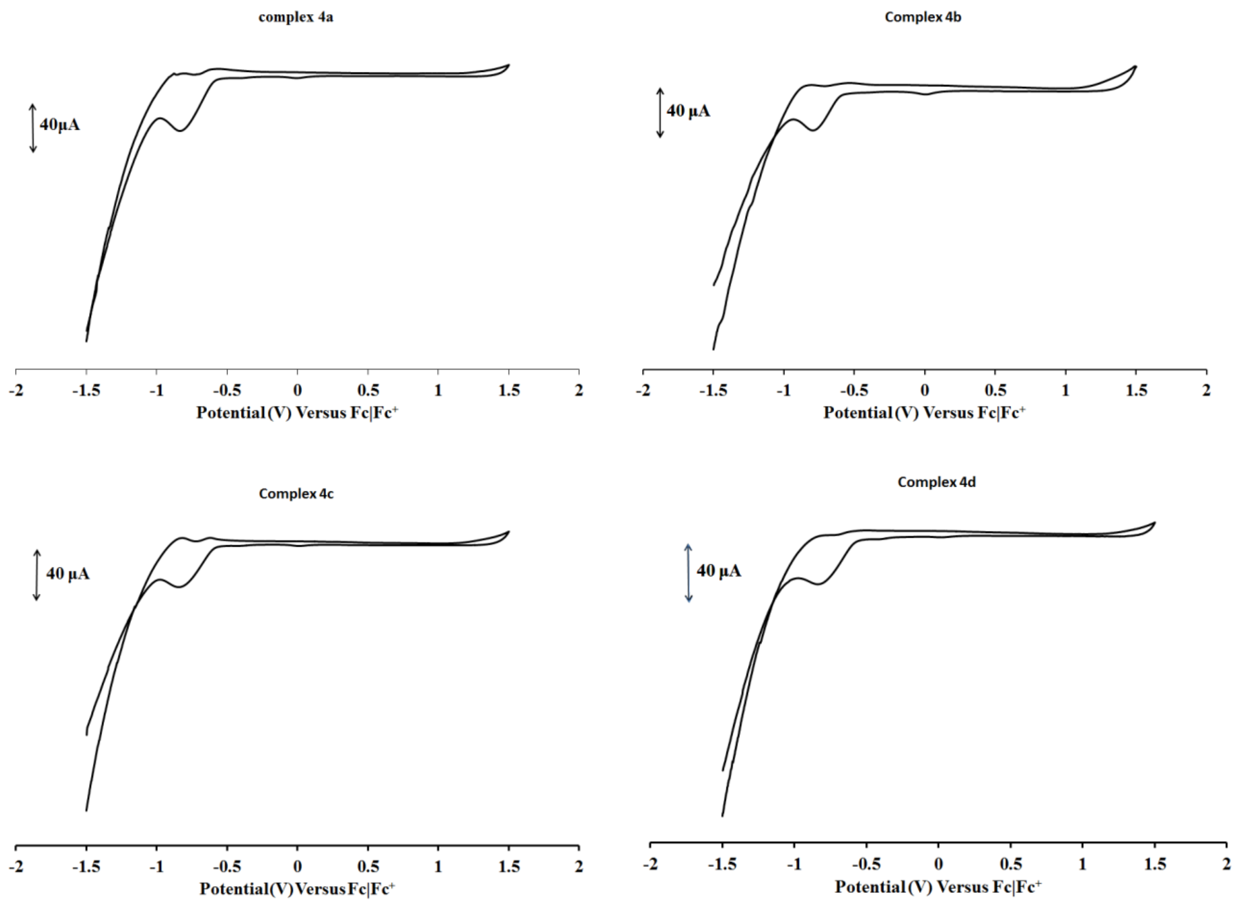

Fig. S3: Cyclic voltammograms of [Ir(**5a-d**)$_3$] showing the quasi-reversible oxidation potential against ferrocene/ferrocenium.

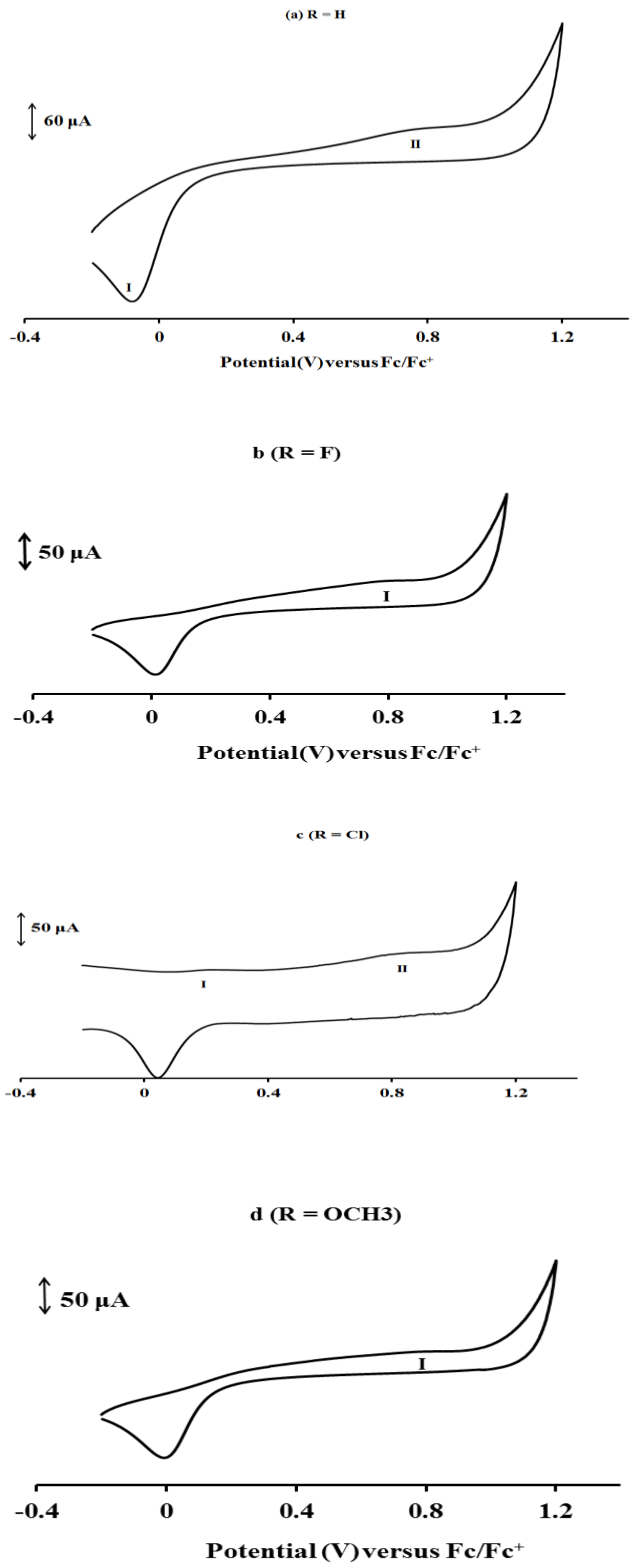

Fig. S4: Cyclic voltammograms of [Ir(**5a-d**)$_3$] showing the reversible reduction potential against ferrocene/ferrocenium.

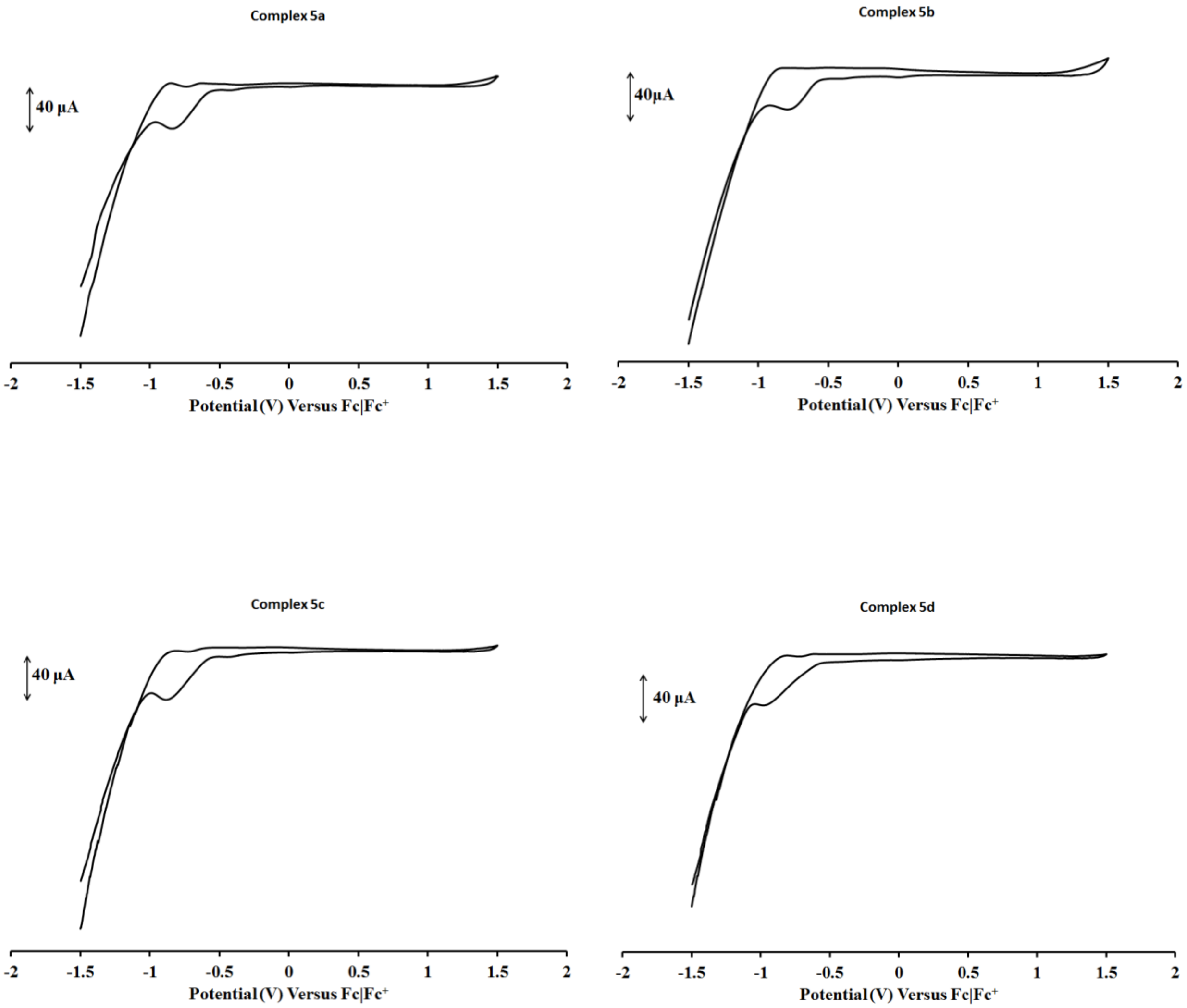

Supplement: Supplementary file 1 [file materials-10-01061-s001.pdf]
